# Reduction of A-to-I RNA editing in the failing human heart regulates formation of circular RNAs

**DOI:** 10.1007/s00395-022-00940-9

**Published:** 2022-06-23

**Authors:** Karoline E. Kokot, Jasmin M. Kneuer, David John, Sabine Rebs, Maximilian N. Möbius-Winkler, Stephan Erbe, Marion Müller, Michael Andritschke, Susanne Gaul, Bilal N. Sheikh, Jan Haas, Holger Thiele, Oliver J. Müller, Susanne Hille, Florian Leuschner, Stefanie Dimmeler, Katrin Streckfuss-Bömeke, Benjamin Meder, Ulrich Laufs, Jes-Niels Boeckel

**Affiliations:** 1 Klinik und Poliklinik für Kardiologie, Universitätsklinikum Leipzig, Liebigstrasse 20, Leipzig, Germany; 2 Institute for Cardiovascular Regeneration, Goethe-University Hospital, Theodor Stern Kai 7, Frankfurt, Germany; 3 German Centre for Cardiovascular Research (DZHK), Partner site RheinMain, Frankfurt, Germany; 4 Institute of Pharmacology and Toxicology, Versbacher-Str. 9, Würzburg, Germany; 5 Heartcenter – Clinic for Cardiology and Pneumology, University Medicine Goettingen, Robert-Koch-Str. 40, Göttingen, Germany; 6 German Centre for Cardiovascular Research (DZHK), Partner site Göttingen, Göttingen, Germany; 7 Department of General and Interventional Cardiology/Angiology, Ruhr University of Bochum, Heart-and Diabetes Center North Rhine-Westphalia, Bad Oeynhausen, Germany; 8 Helmholtz Institute for Metabolic, Obesity and Vascular Research (HI-MAG) of the Helmholtz Zentrum München at the University of Leipzig and University Hospital Leipzig, Leipzig, Germany; 9 Department of Internal Medicine III, University of Heidelberg, Heidelberg, Germany; 10 German Centre for Cardiovascular Research (DZHK), Partner site Heidelberg, Heidelberg, Germany; 11 Heart Center Leipzig at University of Leipzig and Leipzig Heart Institute, Leipzig, Germany; 12 Department of Internal Medicine III, University of Kiel, Kiel, Germany; 13 German Centre for Cardiovascular Research (DZHK), partner site Hamburg/Kiel/Lübeck, Kiel, Germany

**Keywords:** Heart failure, Adenosine-to-inosine RNA editing, Splicing, Circular RNA

## Abstract

Alterations of RNA editing that affect the secondary structure of RNAs can cause human diseases. We therefore studied RNA editing in failing human hearts. Transcriptome sequencing showed that adenosine-to-inosine (A-to-I) RNA editing was responsible for 80% of the editing events in the myocardium. Failing human hearts were characterized by reduced RNA editing. This was primarily attributable to Alu elements in introns of protein-coding genes. In the failing left ventricle, 166 circRNAs were upregulated and 7 circRNAs were downregulated compared to non-failing controls. Most of the upregulated circRNAs were associated with reduced RNA editing in the host gene. ADAR2, which binds to RNA regions that are edited from A-to-I, was decreased in failing human hearts. In vitro*,* reduction of ADAR2 increased circRNA levels suggesting a causal effect of reduced ADAR2 levels on increased circRNAs in the failing human heart. To gain mechanistic insight, one of the identified upregulated circRNAs with a high reduction of editing in heart failure, AKAP13, was further characterized. ADAR2 reduced the formation of double-stranded structures in AKAP13 pre-mRNA, thereby reducing the stability of Alu elements and the circularization of the resulting circRNA. Overexpression of circAKAP13 impaired the sarcomere regularity of human induced pluripotent stem cell-derived cardiomyocytes. These data show that ADAR2 mediates A-to-I RNA editing in the human heart. A-to-I RNA editing represses the formation of dsRNA structures of Alu elements favoring canonical linear mRNA splicing and inhibiting the formation of circRNAs. The findings are relevant to diseases with reduced RNA editing and increased circRNA levels and provide insights into the human-specific regulation of circRNA formation.

**Supplementary Information:**

The online version contains supplementary material available at 10.1007/s00395-022-00940-9.

## Introduction

Heart failure (HF) is characterized by high morbidity, mortality and increasing prevalence [50, 63, 78]. Important genetic and epigenetic contributors of HF have been identified [11, 24, 58]. However, epi-transcriptomic mechanisms that may play an important role during the pathogenesis of HF are yet to be fully explored in the human heart [22, 43, 77].

Adenosine-to-inosine (A-to-I) RNA editing is a post-transcriptional modification process that enlarges transcriptome variety without altering the genome [20]. This modification is the most abundant form of RNA editing in various organisms and is mainly processed by two catalytically active RNA-binding proteins (RBP)—adenosine deaminase acting on RNA 1 (ADAR1) and ADAR2 [2]. ADAR enzymes specifically target double-stranded RNA (dsRNA). After deamination of adenosine, the resulting nucleotide inosine is read and translated as guanosine, thereby impacting both the sequence and the secondary structure of the RNA [68]. A-to-I RNA editing is likely to contribute to the regulation of gene expression, RNA stability and splicing [32, 39, 59, 62, 71]. Changes in A-to-I RNA editing have been associated with different pathological processes in humans. The extent of RNA editing, the underlying mechanisms and potential consequences in the healthy and failing human heart remain unclear.

Transcriptome-wide analysis has shown that A-to-I RNA editing primarily occurs in intronic regions of protein-coding genes and particularly in repetitive Alu sequences, which represent approximately 10% of the human genome [12]. Alu sequences are conserved in primates [28]. After transcription, proximal inverted Alu elements have the ability to form RNA duplexes that can function as a substrate for the dsRNA specific ADARs [13]. Notably, dsRNA stem-loop structures formed by inverted repeated Alu (IRAlus) elements have been associated with the formation of circular RNA (circRNA) [34, 48, 83]. CircRNAs are formed through an alternative splicing mechanism called “back-splicing” and can contribute to human pathologies including HF [1, 4, 5, 19, 51]. In several species, circRNAs are mainly derived from protein-coding genes, but evolutionary conservation is rather found on the gene level than the precise genomic sequence [16, 26]. Although different mechanisms regulating circRNA formation have been proposed, its origin at the species-specific level is not well understood. In this study, we describe a novel primate-specific mechanism, which controls circRNA formation in human hearts. We analyze cardiac transcriptome-wide RNA editing, the expression of ADARs, as well as the association of circRNA expression with HF in humans. We propose a novel ADAR2-mediated process of circRNA formation depending on primate-specific Alu elements.

## Methods

### Patient inclusion and sampling

Patient inclusion for the present study was approved by the ethics committee (appl. no. S-390/2011) [54]. The study was conducted in accordance with the Declaration of Helsinki. All participants have given written informed consent to allow for molecular analysis. The HF group included patients with dilated cardiomyopathy (DCM) or ischemic cardiomyopathy (ICM). The diagnosis of DCM was confirmed after excluding coronary artery disease by coronary angiography. Valvular heart disease was excluded by cardiac magnetic resonance imaging and echocardiography, and myocarditis/inflammatory DCM were determined by histopathology [60]. Patients with a history of uncontrolled hypertension, myocarditis, regular alcohol consumption, illicit drug use, or cardiotoxic chemotherapy were excluded. Patients with normal cardiac function and without transplant rejection were included as controls according to the protected health information (45 C.F.R. 164.514 e2) (bioserve) and the BCI informed consent F-641-5 (biochain). H&E slides were examined to exclude pathological changes.

### Processing of human samples for sequencing

Biopsies of 1–2 mm diameter were immediately washed in ice-cold saline (0.9% NaCl) and transferred and stored in liquid nitrogen until DNA and RNA was extracted. After diagnostic workup of the biopsies (histopathology), the remaining material was evenly dissected. DNA and RNA was extracted using the Allprep Kit (Qiagen) according to the manufacturer’s protocol. RNA purity and concentration were determined using the Bioanalyzer 2100 (Agilent Technologies) with the Eukaryote Total RNA Pico assay for RNA from biopsies.

### RNA and DNA sequencing and analysis

RNA-sequencing libraries were generated using the TruSeq Stranded Total RNA kit (Illumina) according to the standard protocol of the kit. Fastq-reads from paired-end sequencing (RNA and DNA) were generated using CASAVA v.1.82 (Illumina). Mapping was done against the human reference genome (GRCh38) using HISAT2-2.1.0, followed by transcript assembly using StringTie-1.3.3b. Individual GTF files with gene and transcript abundances per sample were then merged by applying StringTie with the ‘-e’ option. Finally, read count and transcript count information was generated using StringTie prepDE.py script. For genome sequencing, 1 µg of total gDNA was sheared using the CovarisTM S220 system, applying two treatments of 60 s each (peak power = 140; duty factor = 10) with 200 cycles/burst. 500 ng of sheared gDNA was taken and whole-genome libraries were prepared using TruSeq DNA sample preparation kit according to manufacturer’s protocol (Illumina, San Diego, US). Mapping was done against the human reference genome (GRCh38) using HISAT2-2.1.0. Further processing and variant calling was done following “best-practice-guidelines” of the Genome Analysis Toolkit (GATK) (https://gatk.broadinstitute.org/hc/en-us/articles/360035535932-Germline-short-variant-discovery-SNPs-Indels-).

### RNA editing analysis

FASTQ files were used to identify and characterize RNA editing events in the transcriptome using the RNAEditor tool [35]. The output of the analysis excluded known SNPs and sequencing errors but included detailed information about each editing site, such as gene names, segments, number of total reads, number of edited reads, and the ratio of RNA editing and most importantly information on the type of RNA editing. Additionally, a summary file in BED-format was generated, which showed the sum of editing sites per segment for each gene. The summary also included features such as length, number of editing sites, and editing ratio. The identified editing events summarized in the obtained BED files were used to inspect the identified editing events. BED files were loaded into the Integrative Genomics Viewer (IGV) for first validation by comparing the summarized obtained editing events with the amount of RNA editing on single RNA base level [61].

### Analysis of RNA editing overlaps in BSS-flanking introns

All genomic coordinates of up- and downstream introns flanking the back-splice site (BSS) of regulated circRNAs (*n* = 173) were extracted using UCSC genome browser. Scores of putative RNA editing events were overlapped with given coordinates to assess, whether RNA editing events were indeed found in flanking introns of regulated circRNAs BSS in HF patients (*n* = 19) and controls (*n* = 10).

### CircRNA analysis

The rRNA‐depleted RNA-sequencing data were analyzed using an established circBase pipeline on default settings with an anchor length of 20 bp as done before in endothelial cells [25]. The circBase pipeline identified potential circRNAs from reads that were not mapped to the reference genome. Results were filtered for circRNAs having at least two unique reads, while the mapping quality was set greater than 35 bp for both anchor sequences, and to a length shorter than 100,000 bp. The existence of the predicted circRNAs was verified by searching the raw sequencing data with 80 bp of the back‐spliced sequences. To finally annotate the identified circRNAs the program HOMER was used. RNA-seq data were analyzed using circBase [25, 55].

### Homogenization and lysis of heart tissue for RNA analysis

Heart tissue (50 mg) was washed 3 times with ice-cold DPBS (17-512F, Lonza, CH). The tissue was placed in 700 µL QIAzol lysis reagent. For homogenization, heart tissue was added to the Tissue Homogenizing CKMix (KT03961-1-009.2, Thermo Scientific, US) and processed in the Precellys 24 Tissue Homogenizer (P000669-PR240-A, Bertin instruments, FR) at 5000 rpm, 4 °C until the tissue was fully homogenized. Lysate was centrifuged at 14,000 rpm, 4 °C for 10 min. RNA was extracted from the supernatant using the miRNeasy Mini Kit (217004, Qiagen, DE) with on-column DNase digestion according to manufacturer’s instructions.

### Cell culture and siRNA-mediated knockdown of ADAR2

Primary human cardiomyocytes (HCM) were cultured in Myocyte Basal Medium (C-22270) supplemented with Growth Medium Supplement Pack (C-39270) purchased at Promocell, DE. HEK-293 cells were cultivated in DMEM/F12 medium (Gibco, UK) supplemented with 10% FCS, 1% Penicillin/Streptomycin mix and 1% glutamine (all Gibco, UK). Cells were cultured at 37 °C, 20% O_2_ and 5% CO_2_ conditions and were split when they reached approximately 70% confluency. One day before transfection, 2 × 10^5^ HCM were plated on a 60 mm tissue culture plate. siRNA was transfected using the Gene Trans II Transfection Reagent (0202B, MoBiTec) with a final concentration of 60 nM in Opti-MEM I serum reduced medium (31985070, Thermo Scientific) according to manufacturer’s instruction. After 4 h, cells were transferred to full growth medium and harvested after a total of 24 h or 48 h. The following siRNA sequences were used for lipofection Scr: UCUCUCACAACGGGCAUUU[dT][dT], siADAR1: GCUAUUUGCUGUCGUGUGA[dT][dT], siADAR2: GAUCGUGGCUUGCAUUAA [dT][dT]. siRNA was produced and predesigned by Merck, DE.

### Generation of iPSC-CM, AAV-mediated ADAR2 knockdown and AAV-mediated circAKAP13 overexpression

Human-induced pluripotent stem cell-derived cardiomyocytes (hiPSC-CM) were generated as described before [6]. Cardiac differentiation of hiPSCs using standardized protocols, including cardiac mesoderm induction by subsequent activation and inhibition of the WNT pathway [47] and metabolic selection [75] were described earlier [6]. Cells were studied 60 days after initiation of differentiation. After differentiation, purity of hiPSC-CMs was determined by flow cytometry analysis (> 90% cardiac TNT +) or by morphology. HiPSC-CMs were maintained in RPMI 1640 supplemented with Glutamax, Hepes and B27 supplement. Fully differentiated hiPSC-CMs were transduced with AAV6 particles. An increasing multiplicity of infection (MOI) of 10^2^–10^4^ was used for transduction with control AAV6-EGFP and AAV6-circAKAP13^IR−Alu^ which were generated as described before [37]. A MOI of 10^5^ was used for the control AAV6-shscrambled (AAV6-GFP-U6-shRNA, 7043, Vector Biolabs) and the AAV-ADARB1-shRNA (AAV6-GFP-U6-h-ADARBI-shRNA, shAAV-200407, Vector Biolabs).

### Mini-gene splicing assay

One day before transfection, 6.6 × 10^5^ HEK293 cells were plated per well in a 6-well tissue culture plate. Cells were transfected with polyethylenimine (PEI) reagent (23966-1, PolyScience, US) in a ratio of DNA:PEI 1:3 in Opti-MEM. After 4 h, cells were re-incubated in growth medium and harvested after 24 h. HEK293 cells were transfected with either ADAR1 (pmGFP-ADAR1-p110, Addgene, US), ADARB1 (RC209073L1, Origene, US) and PS100064 (Origene, US) or the *circAKAP13* mini-genes and pcDNA3.1( +) plasmids.

### Quantitative real-time polymerase chain reaction (qRT-PCR)

Total RNA was extracted from lysed cells using the miRNeasy Mini Kit (217004, Qiagen, DE) with on-column DNase digestion according to manufacturer’s instructions. 30 µL total RNA were eluted and 1 µg was reverse transcribed into cDNA using the following reagents at final concentrations: 2.5 U/µL M-MLV Reverse Transcriptase (28025021, Thermo Scientific, US), 0.25 mM dNTP mix (10297018, Thermo Scientific, US), 5 µM Random Hexamer Primers (SO142, Thermo Scientific, US) and 1 U/µL RiboLock RNase Inhibitor (EO0381, Thermo Scientific, US). Generated cDNA was diluted to a working concentration of 5 ng/µL and qRT-PCR was performed with PowerUp SYBR Green Mastermix (A25742, Thermo Scientific, US) according to manufacturer’s instruction. Relative gene expression was calculated with the 2^−∆Ct^ or the ∆∆Ct method using *RPLP0* as a reference gene. The primer sequences are listed in Supplementary Table I.

### Human tissue panel

Total artery (HR-810), atrium (left, R1234126-50), brain (R1234035-50), kidney (R1234142-50), liver (R1234149-50), lung (R1234152-50), skeletal muscle (R1234171-50), stomach (R1234248-50), ventricle (R1234138-50) were purchased at Amsbio LLC, UK, quantified with the Denovix DS-11 spectrophotometer and 1 µg was transcribed into cDNA as described above. Generated cDNA was amplified with qRT-PCR and analyzed by gel electrophoresis.

### Oligo(dT)-selection

Total RNA was extracted from lysed cells as described above. 5 µg of total RNA was selected with Oligo d (T)_25_ magnetic beads (S1419S, NEB, US). Buffers were prepared as recommended by the manufacturer. After loading of RNA on magnetic beads, the mixture was incubated for 10 min. The supernatant was then analyzed as the oligo(dT)- negative fraction. Bound RNA was washed 3 times and then eluted as the oligo(dT)-positive fraction. RNA fractions were synthesized into cDNA as described above and analyzed by KOD XTreme HotStart PCR according to manufacturer’s instructions.

### RNase R digestion

Total RNA (5 µg) was digested with 1 U/µg RNase R (RNR07250, Epicentre, US) for 10 min at 37 °C and 600 rpm on a thermo shaker. RNase R digestion was stopped at 95 °C for 3 min. RNA was transcribed into cDNA as described above and further analyzed with KOD Xtreme HotStart Polymerase PCR.

### Nucleus/cytoplasm fractionation

A lysis buffer containing 10 mM (pH = 8.0) Tris–HCl (11814273001, Merck, DE) containing 140 mM NaCl (S6546, Merck, DE), 1.5 mM MgCl_2_ (63069, Merck, DE) and 0.05% NP-40 was prepared. Cells were harvested in ice-cold D-PBS (Lonza, CH) and centrifuged at 300 *g* and 4 °C for 10 min. The pellet was re-suspended in lysis buffer with RNase inhibitor and incubated on ice for 5 min. After another centrifugation at 1000 *g* and 4 °C for 3 min, the supernatant was mixed with 700 µL QIAzol and processed as the cytoplasmic fraction. The remaining cell pellet was re-suspended in 700 µL QIAzol and processed as the nucleus fraction. Total RNA from both fractions was extracted and cDNA was synthesized as described above. Analysis of fractions was performed with KOD HotStart Xtreme Polymerase according to manufacturer’s instructions.

### Immunoblotting

For immunoblotting of tissue samples, 50 mg heart tissue was washed 3 times with ice-cold DPBS (17-512F, Lonza, CH). For homogenization, heart tissue was added to the Tissue homogenizing CKMix (KT03961-1-009.2, Thermo Scientific, US) and processed in the Precellys 24 Tissue Homogenizer (P000669-PR240-A, Bertin instruments, FR) at 5000 rpm and 4 °C until the tissue was fully homogenized. The lysate was centrifuged at 14,000 rpm, 4 °C for 10 min. For immunoblotting of cell samples, HCM cells were lysed in RIPA buffer containing 1X HALT protease/phosphatase inhibitor cocktail (78429, Thermo Scientific, US). Proteins were loaded on a 10% SDS–Polyacrylamide gel. SDS-PAGE was performed in the Mini-PROTEAN System (Biorad, US) and proteins were blotted on a 0.45 µm nitrocellulose membrane (Biorad, US). The membrane was blocked in 5% non-fat dry milk (in TBS-T) and incubated with antibodies directed against ADAR1 (1:1000, D7E2M, Cell Signaling, US), ADARB1 (1:500, SAB1405426, Thermo Scientific, US) and ß-actin (1:5000, 1829b, Abgent, US) as a loading control. Densiometric analysis was performed with ImageJ.

### RNA immunoprecipitation

A total of 3.7 × 10^6^ HEK293 cells each were seeded on 4 × 100 mm tissue culture plates. After 24 h, each plate was transfected with 1800 ng ADAR2 using the PEI-method as described above. After another 24 h cells were harvested and pooled. RNA immunoprecipitation (RIP) was performed with the Magna-RIP RNA-Binding Protein Immunoprecipitation Kit (17-700, Merck, DE) according to manufacturer’s instructions. RIP was performed with normal rabbit IgG (3.5 µg, RIgG, Nordic-MUbio, NLD) and rabbit anti ADARB1 (3.5 µg, 22248-1-AP, Proteintech, US) antibodies. IP was functionally validated by immunoblotting using the same antibodies as described above. RNA bound to ADAR2 was reverse transcribed and tested with qRT-PCR as described above.

### Cloning, Sanger sequencing and mini-gene construction

For the construction of the *circAKAP13* mini-genes, the *circAKAP13* sequence as well as sequences of Alu flanking the back-splice site (either 5’AluSx3 and 3’AluSz, 5’AluSz and 3’AluSz or no flanking Alu elements) were amplified with KOD Xtreme HotStart PCR (71975, Merck, DE) according to manufacturer’s instructions. PCR products were visualized with GelStar Nucleic Acid Gel Stain (50535, Lonza, CH) using agarose gel electrophoresis and bands with the expected size were purified with Monarch DNA Gel Extraction Kit (T1020S, NEB, US). Purified PCR products were cloned into a linearized pcDNA3.1( +) vector using InFusion HD Cloning Kit (102581, Takara, US). Plasmids were purified with Monarch Plasmid Miniprep Kit (T1010S, NEB, US) and the insert was validated with Sanger sequencing. Primers are listed in Supplementary Table I. For PCR product cloning, desired targets were amplified and purified with KOD Xtreme HotStart PCR as described above. Purified PCR products were cloned into pMinit2.0 vector using NEB PCR Cloning Kit (NEB, US) according to manufacturer’s instructions. Purified plasmids were analyzed by Sanger sequencing to confirm PCR product amplification and sequences were screened for RNA editing using UCSC genome browser’s BLAT.

### Northern blotting

For the preparation of RNA probes, 5 µg of template DNA amplified with DIG-tag primers (Supplementary table I) were transcribed with T7 polymerase from the DIG RNA labeling kit (11175025910, Roche, CH) for 2 h at 37 °C. Residual DNA was digested with DNase I for 15 min at 37 °C. DIG-labeled RNA probes were isolated using phenol–chloroform extraction and eluted in 25 µL EB (19086, Qiagen, DE). For northern blotting, 8 µg of RNA were loaded on a 1.2% agarose gel in 1X MOPS buffer (AM8671, Thermo Scientific, US) and RNA was separated by size through electrophoresis at 100 V. After electrophoresis, agarose gel was blotted onto positively charged nylon membrane (11209299001, Roche, CH). The RNA was then UV cross-linked to the membrane and pre-hybridized for 30 min at 68 °C with ultrasensitive hybridization buffer (AM8670, Thermo Scientific, US). The membrane was incubated with RNA probes at 50 ng/mL hybridization buffer over night at 68 °C. The membrane was then washed with high and low stringency wash buffer prior to blocking in 1X blocking solution from DIG wash and block buffer set (11585762001, Roche, CH). After blocking, membrane was incubated with 1:5000 Anti-DIG AP from DIG Luminescent Detection Kit (11363514910, Roche, CH). CSPD was used as a substrate for chemiluminescent detection.

### Data availability

Sequencing data generated in this study and all other data supporting the findings of this study are available from the corresponding author upon reasonable request. Source data are provided with this paper.

### Code availability

The RNAEditor tool used for RNA editing analysis is available from http://rnaeditor.uni-frankfurt.de/. All codes used in this study are publicly available.

### Statistical analysis

First, sample values were tested for Gaussian distribution. If the normality test was passed, significance was tested with the student’s *t*-test for comparison of two groups. If more than two groups were compared, the one-way ANOVA with Tukey’s post hoc correction was performed. If the samples did not pass the normality test, two unpaired groups were analyzed using the Mann–Whitney *U* test and paired groups were analyzed using Wilcoxon matched-pairs signed-rank test. More than two groups were compared with the Kruskal–Wallis test. For correlation analysis, groups were first tested for Gaussian distribution. If the normality test was passed, Pearson correlation was performed, otherwise, Spearman correlation analysis was performed. *P* values under 0.05 were considered significant. Statistical analysis was performed using GraphPad Prism 8.

## Results

### Transcriptome-wide analysis of RNA editing in the human heart

**Fig. 1:**
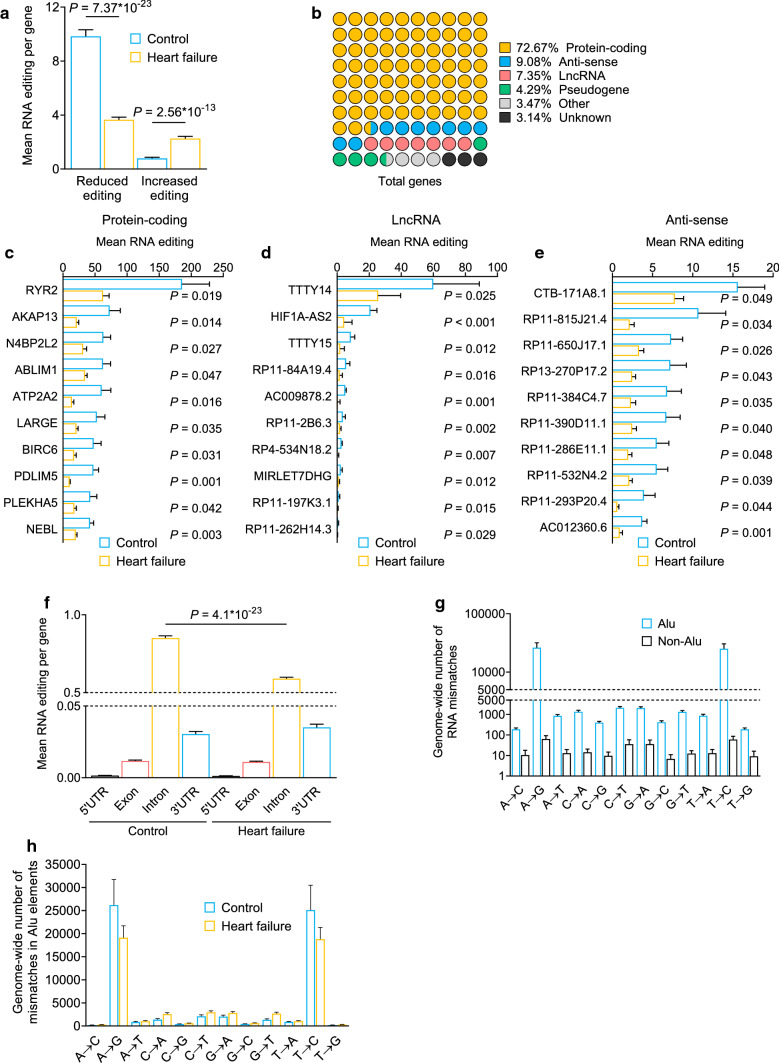
RNA editing in human hearts. **a** Mean RNA mismatching events per gene, divided into genes with reduced and increased editing in failing human hearts (yellow, *n* = 20) compared to controls (blue, *n* = 10). **b** Biotypes of 1211 genes with significantly different RNA editing levels. **c** Significant reduction of mean editing rates in protein-coding genes, **d** lncRNAs, and **e** anti-sense transcripts. **f** Mean RNA editing events in different regions of genes in HF patients (*n* = 20) and controls (*n* = 10). **g** Genome-wide RNA editing events located in Alu elements or non-Alu sequences in controls (*n* = 10). RNA editing was grouped by base substitution. **h** Genome-wide RNA editing events in repetitive Alu sequences in HF patients (*n* = 20) and controls (*n* = 10). RNA editing was grouped by base substitution. A-to-G and T-to-C mismatches are indicative of A-to-I RNA editing. Graphs are shown as mean + SEM. Statistical differences were calculated using Student’s *t*-test

To investigate the extent of RNA editing in HF, we performed transcriptome sequencing from left ventricle tissue from failing and non-failing hearts and identified RNA editing events. RNA editing events were detected in the nucleotide sequence of 1211 genes. Considering all genes with differential editing in failing left ventricles, we found the absolute decrease in RNA editing events to be more profound compared with the increase (Fig. 1a and Supplementary Fig. 1a). The editing events were primarily located in RNA transcripts encoding for protein-coding genes (Fig. 1b and c and Supplementary Fig. 1b), lncRNAs (Fig. 1d and Supplementary Fig. 1c) and in natural anti-sense transcripts (Fig. 1e and Supplementary Fig. 1d). The RNA editing observed in different functional elements of protein-coding genes predominantly appeared in intronic elements. In failing hearts, RNA editing was decreased within introns, whereas RNA editing in 3’ untranslated regions (UTR), exons and 5’UTR was not changed (Fig. 1f). The majority of the RNA editing events in the human heart were located in Alu elements (Fig. 1g and Supplementary Fig. 1e), while A-G and T-C nucleotide switches were the most prominent RNA base changes. Both code for A-to-I editing, which made up more than 80% of all detected RNA editing events and were reduced in HF patients (Fig. 1h and Supplementary Fig. 1f–n). Together, these data show that A-to-I RNA editing is the primary type of RNA editing in the human heart and is mainly found in Alu elements that are located in introns of protein-coding genes*.*

### CircRNA levels are increased in failing hearts

**Fig. 2:**
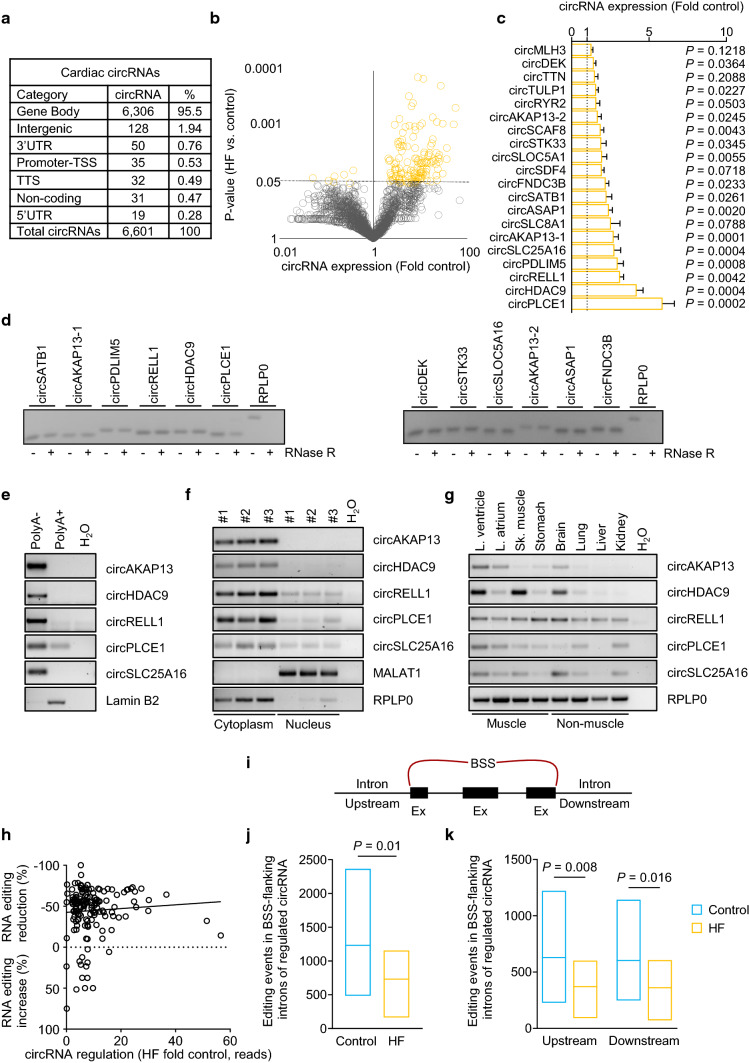
Regulation and characterization of circRNAs in failing human hearts. **a** Genetic origin of the identified cardiac circRNAs based on biotype annotations by HOMER. **b** Volcano plot showing HF-regulated circRNAs in heart tissue samples of HF patients (*n* = 20) and controls (*n* = 10). Dashed line indicates *P* = 0.05. **c** Regulation of identified circRNA candidates using qRT-PCR. Top circRNAs regulated in HF identified via NGS were validated using qRT-PCR with specific primer pairs targeting the BSS in heart tissue of controls (*n* = 5) and HF patients (*n* = 18). **d** Sensitivity of circRNA transcripts to digestion with exonuclease RNase R (1 U/µg RNA) using total RNA isolated from human heart tissue. The linear mRNA of *RPLP0* served as a positive control. **e** CircRNA enrichment in PolyA(-) and PolyA( +) fractions, as bound to oligo-(dT)-beads using total RNA obtained from primary human cardiomyocytes (HCM). **f** Cellular localization of circRNA candidates in cytoplasmic- and nuclear-enriched fractions of human cardiomyocytes. *MALAT1* was used as a control for the nuclear and *RPLP0* as a control for the cytoplasmic fraction. **g** CircRNA expression in human total RNA tissue panel. RNA was grouped by muscle and non-muscle tissues. *RPLP0* served as a reference gene. **h** RNA editing events in the host gene compared to the regulation of the related circRNAs in the failing human heart. **i** Schematic overview of editing screening located in flanking introns. Introns up- and downstream of the BSS (red bracket) of regulated circRNAs (*n* = 173) were screened for RNA editing events. **j** Min–Max plot of overlapping RNA editing events in flanking introns and **k** divided by up- and downstream localization of the regulated circRNA in HF patients (*n* = 19) and controls (*n* = 10). Line indicates mean. All bar graphs show mean + SEM. Statistical differences were calculated using Student’s *t*-test

RNA editing in introns of protein-coding genes has been associated with circRNA formation [32]. Therefore, we analyzed whether the loss of RNA editing in introns of protein-coding genes might be related to changes in circRNAs in failing hearts. We identified 6,601 transcribed circRNAs in the left ventricle. The majority (95.5%) of circRNAs consisted of exons and introns of parental protein-coding genes (Fig. 2a and Supplementary Fig. 2a). A total of 166 circRNAs were upregulated in the failing left ventricle compared to controls, while 7 were downregulated (Fig. 2b). Importantly, the majority of circRNAs (109) were increased despite normal mRNA levels of their host genes, suggesting an alternative splicing mechanism underlying the accumulation of these circRNAs (Supplementary Fig. 2b–l).

To confirm our identification of circRNAs, we analyzed 20 circRNAs from control hearts (*n* = 5) and HF patients (*n* = 18) using quantitative real-time PCR (qRT-PCR) with primers spanning over the circRNA-specific back-splice site (BSS) (Fig. 2c) [29]. This showed a validation for the increase of circRNA level for the majority of the selected candidates (15 out of 20). Furthermore, we selected 12 circRNAs based on their regulation and tested their circularity by analyzing their resistance to the 3′–5′ exoribonuclease RNase R. All 12 transcripts were resistant to RNase R, whereas the linear Ribosomal Protein Lateral Stalk Subunit P0 (*RPLP0*) mRNA was not (Fig. 2d), suggesting that the identified transcripts indeed represent circular RNAs. Of these, 5 circRNAs were selected for further characterization, namely *circPLCE1*, *circHDAC9*, *circRELL1*, *circSLC25A16,* and *circAKAP13*. The PCR products from the circRNA candidates were analyzed using Sanger sequencing to validate the existence of covalently closed back-splice sequences (Supplementary Fig. 2c–f). All 5 circRNAs were enriched in the poly-A-negative and cytoplasmic fractions (Fig. 2e and f). Together, these control experiments confirm the circular structure of the shortlisted circRNAs.

To investigate a potential tissue enrichment, the expression of all 5 circRNA candidates was determined in different human tissue samples (Fig. 2g). This showed that all 5 circRNA candidates are expressed in the left ventricle of the human heart. Finally, we assessed the extent of RNA editing in the host genes of all upregulated circRNA candidates. A reduction in RNA editing along with a concomitant increase in circRNA levels was evident for the vast majority of host genes in failing hearts (Fig. 2h). We analyzed RNA editing events in introns flanking the BSS of HF-regulated circRNAs (Fig. 2i). We found a reduction of RNA editing events in upstream and downstream introns flanking the circRNA BSS of HF patients (Fig. 2j and k). Together, these data confirmed the increase of bona fide circRNAs in association with a decrease in RNA editing.

### The ADAR editing enzymes are differentially expressed in failing human hearts

**Fig. 3:**
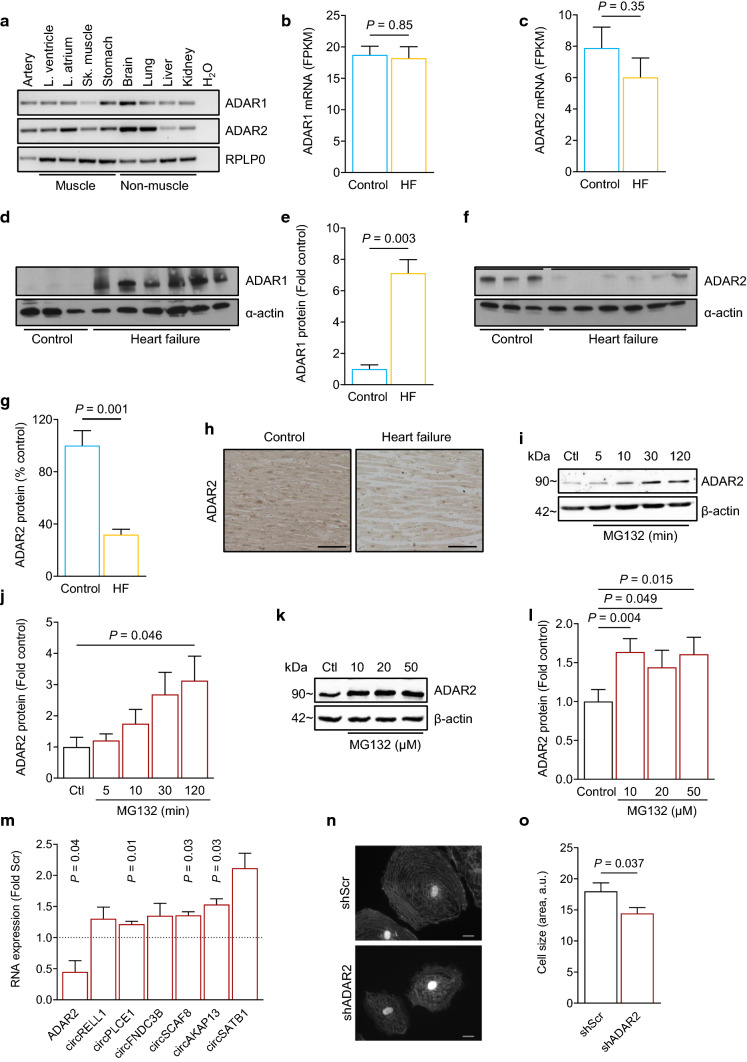
Expression and regulation of ADAR1 and ADAR2 enzymes. **a** *ADAR1* and *ADAR2* expression in human total RNA tissue panel. **b** *ADAR1* and **c** *ADAR2* mRNA expression obtained from NGS data of controls (*n* = 10) and HF patients (*n* = 20). **d** Immunoblot of ADAR1 and **e** quantitation of ADAR1 and **f** immunoblot of ADAR2 and quantitation of **g** ADAR2 protein expression in human heart tissue of controls (*n* = 3) and HF patients (*n* = 6) normalized to α-actin. **h** ADAR2 staining in paraffin-embedded human control and failing myocardium. Detected with DAB in 20 × magnification. Scale bar indicates 100 µm. **i** Immunoblot of human cardiomyocytes treated with MG132 for increasing amounts of time. Cells were treated with 20 µM MG132 for 5, 10, 30, or 120 min. **j** Quantitation of ADAR2 protein expression in human cardiomyocytes treated with MG132 for increasing amounts of time normalized to ß-actin (*n* = 3). **k** Immunoblot of ADAR2 in human cardiomyocytes treated with either 10, 20 or 50 µM MG132 for 2 h. **l** Quantitation of ADAR2 protein expression in human cardiomyocytes treated with increasing concentrations of MG132 normalized to ß-actin (*n* = 5). **m** RNA expression of circRNA candidates after siADAR2 knockdown. Human cardiomyocytes were transfected and RNA expression was analyzed with qRT-PCR after 24 h (*n* = 5–7). **n** ADAR2-dependent effects in human iPSC-CMs were analyzed using AAV6-mediated shRNA knockdown of ADAR2. Representative images after immunofluorescence staining of the sarcomeric protein α-actinin. Scale bar = 20 µm. **o** Analysis of cell size after shADAR2 in human iPSC-CMs. *n* = 51 random images per condition and experiment (3). All graphs show mean + SEM. Statistical differences were calculated using Student’s *t*-test. *Ctl* indicates Control, *Scr* indicates scrambled

To elucidate the potential cause for the loss of RNA editing and the increase of circRNAs in the failing heart, we analyzed the expression of the two catalytically active members of the ADAR family, namely ADAR1 and ADAR2. Both *ADAR1* and *ADAR2* were ubiquitously expressed in human tissues at the mRNA level and were not transcriptionally regulated in the failing heart (Fig. 3a–c). On the protein level, ADAR1 was increased (Fig. 3d and e and Supplementary Fig. 3a and 3b), whereas ADAR2 protein was reduced in the failing heart (Fig. 3f–h). Since the ADAR2 protein expression can be regulated at the level of protein stability [53], we treated primary human cardiomyocytes with increasing incubation time or increasing concentrations of the proteasome inhibitor MG132 resulting in an increase of ADAR2 at the protein level (Fig. 3i–l). This suggests that ADAR2 loss in failing hearts is likely due to increased proteasome-mediated degradation. Notably, the loss of ADAR2 in human cardiomyocytes increased circRNA levels, whereas host gene mRNA expression was not changed (Fig. 3m and Supplementary Fig. 3c). However, the overexpression as well as the knockdown of ADAR1 had no effect on circRNA levels (Supplementary Fig. 3d–f and Supplementary Fig. 3g–i). To analyze the effect of ADAR2 downregulation in human induced pluripotent stem cell-derived cardiomyocytes (hiPSC-CMs), we used an (Adeno-Associated Virus 6) AAV6-mediated shRNA knockdown approach of ADAR2 and found the cell size to be reduced after a knockdown of ADAR2 compared to Scr hiPSC-CMs (Fig. 3n and o). Together, these data reveal a reduction of ADAR2 as a catalytically active A-to-I editing enzyme in the failing human heart contributing to increased circRNA levels.

### A-to-I RNA editing is reduced in intronic Alu elements flanking circRNAs

**Fig. 4:**
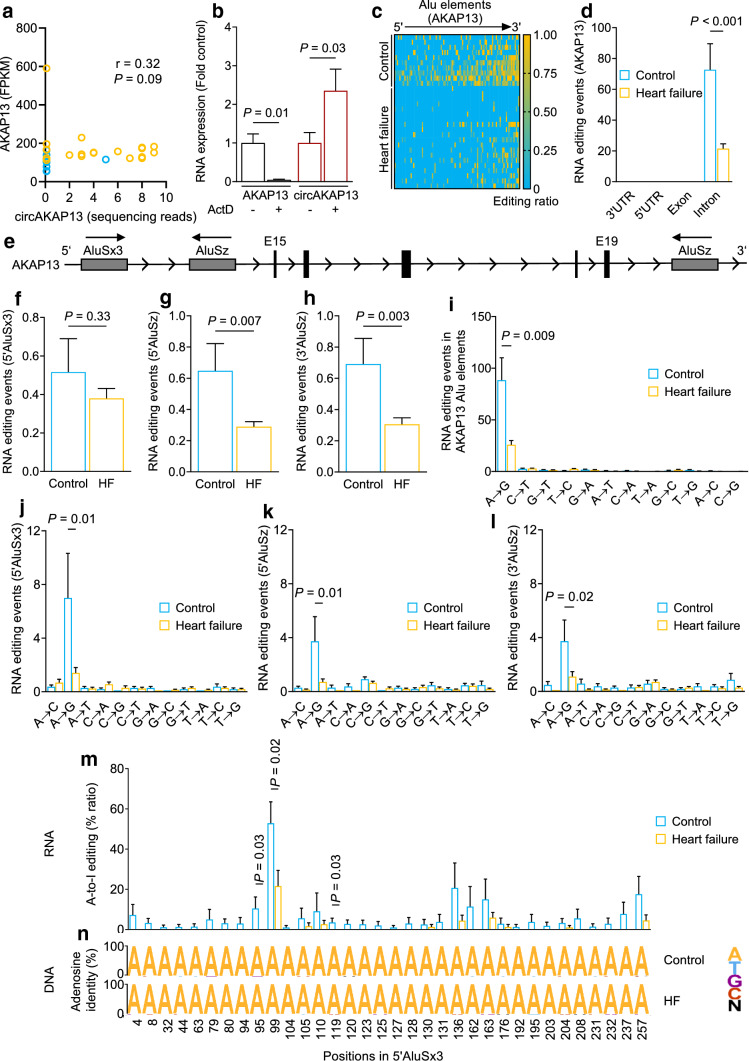
RNA editing in the *AKAP13* gene in the healthy and failing human heart. **a** Spearman correlation of *circAKAP13* expression as BSS spanning reads against the expression of its host *AKAP13* gene. Data are presented as fragments per kilobase of transcript per million mapped fragments (FPKM). **b** RNA stability of *circAKAP13* and its host gene *AKAP13* mRNA. Human cardiomyocytes were treated with 1 µg/mL Actinomycin D for 24 h and *AKAP13* and *circAKAP13* RNA expression was analyzed with qRT-PCR (*n* = 4). **c**, **d** RNA editing in the *AKAP13* gene in heart tissue of controls (*n* = 10) and HF patients (*n* = 20). **c** Heat map of editing sites in Alu elements as editing ratio per sequencing reads. Number of reads with base mismatches was divided by the total number of reads. **d** Localization of editing events in the *AKAP13* gene. **e** Schematic overview of the *AKAP13* gene sequence including the BSS of *circAKAP13* and its flanking intronic Alu elements (encoded on the sense strand). **f–h** Mean RNA editing events in intronic Alu sequences flanking the *circAKAP13* BSS in heart tissue of controls (*n* = 10) and HF patients (*n* = 20). **f** Editing in the AluSx3 and **g** the AluSz flanking the 5’ BSS and **h** the AluSz element flanking the 3’ BSS of *circAKAP13* in the *AKAP13* pre-mRNA. **i** Mean number of nucleotide mismatches in Alu repeats of the *AKAP13* gene in controls (*n* = 10) and HF patients (*n* = 20). All editing events were grouped by base substitution and number of mismatches. **j-l** Mean RNA editing events grouped by their respective nucleotide switch in Alu sequences flanking the intronic region of the *circAKAP13* back-splice site. **j** AluSx3 and **k** AluSz flanking the 5’ BSS and **l** AluSz element flanking the 3’ BSS of *circAKAP13*. **m** Ratio of A-to-I RNA editing events in single nucleotide positions in the 5’ AluSx3 element of the *circAKAP13* BSS. The number of reads with A-to-I base switches was divided by the total number of reads covering the 5’AluSx3 element of the *circAKAP13* BSS. Blue— control (*n* = 10), yellow—HF (*n* = 20). **n** Data obtained from RNA-seq reads was compared to the DNA sequence of the same individuals in control (*n* = 10) and HF patients (*n* = 13) group to exclude potential SNPs in genomic DNA. All bar graphs show mean + SEM. Statistical differences were calculated using Student’s *t*-test or Mann–Whitney test

To gain further mechanistic insights into the impact of RNA editing on circRNA formation, we analyzed the reduction of RNA editing in depth for one exemplary circRNA. The circular RNA of AKAP13 was selected because circAKAP13 (hsa_circ_0104801) was increased both in HF (Fig. 2b and c) and after siRNA-mediated reduction of ADAR2 (Fig. 3m), whereas the host gene AKAP13 was among the most highly regulated protein-coding mRNAs at the level of RNA editing in HF (Fig. 1c). The expression of *circAKAP13* did not correlate with the expression of its host *AKAP13* gene (Fig. 4a and Supplementary Fig. 4a). CircRNAs are considered to have higher transcript stability than their host gene mRNA [5]. Treatment of cardiomyocytes with the transcriptional inhibitor actinomycin D revealed a higher stability of *circAKAP13* compared to its corresponding linear *AKAP13* mRNA transcript (Fig. 4b). This suggests an underlying mechanism resulting in the increase of *circAKAP13* formation independent of the transcriptional levels of *AKAP13* mRNA.

The formation of circRNAs is associated with Alu elements located in introns flanking the BSS. These inverted repeated Alu elements are able to form dsRNA stem-loop structures as a prerequisite for ADAR-mediated RNA editing [42]. These dsRNA stem-loop structures have been previously associated with enhanced circularization of RNAs. Interestingly, RNA editing events in the *AKAP13* RNA transcript were confined to Alu elements (Fig. 4c) located in introns (Fig. 4d). Therefore, we screened for RNA editing in Alu elements up- and downstream of the BSS that is formed by exons 15 and 19 of *AKAP13*. We identified two Alu elements (AluSx3 and AluSz) in intron 14 upstream of the 5’ BSS and one Alu element (AluSz) located downstream of the 3’ BSS in intron 20 of *AKAP13* (Fig. 4e). These three Alu elements are only found in primate genomes (Supplementary Fig. 4b–d). The number of nucleotide mismatches was reduced in both 3’ and 5’ AluSz elements in failing hearts (Fig. 4f–h and Supplementary Fig. 4e) and primarily driven by the reduction of A-to-G mismatches (Fig. 4i–l). Closer analysis of A-to-I RNA transcript editing in the 5’AluSx3 element flanking the *circAKAP13* BSS revealed significant reductions at the adenosine nucleotide positions 95, 99 and 119 in HF patients (Fig. 4m). These reductions were confirmed by next generation genome sequencing of the same patients (Fig. 4n). Together, these data reveal a loss of A-to-I RNA editing in intronic Alu elements flanking the *AKAP13* circRNA BSS in HF patients, which was concomitant with an increase in the corresponding circRNA.

### ADAR2 mediates A-to-I editing and formation of circRNAs

**Fig. 5:**
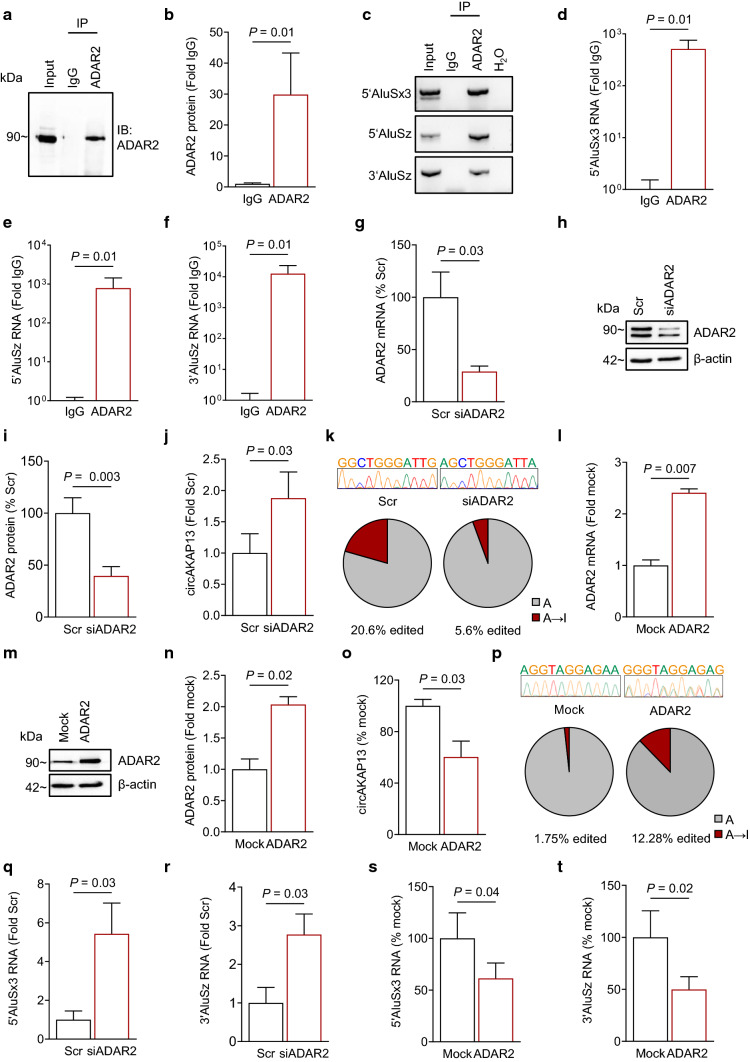
ADAR2-mediated RNA editing in Alu elements and its impact on circRNA formation. **a–f** RNA immunoprecipitation (RIP) of ADAR2 and interacting Alu elements. Immunoprecipitation was **a** validated for ADAR2 and **b** quantified using immuno-blotting (*n* = 3). **c** The immunoprecipitated RNA was analyzed using qRT-PCR targeting all three Alu elements with subsequent agarose gel electrophoresis. Interaction of ADAR2 with **d** 5’AluSx3, **e** 5’AluSz, and **f** 3’AluSz elements was analyzed using qRT-PCR (*n* = 3). Graphs show mean + SEM. Statistical differences were calculated using Mann–Whitney test. SiRNA-mediated knockdown of *ADAR2* in HCM was validated on **g** mRNA (*n* = 5) and on **h** protein level. **i** Quantitation of ADAR2 protein expression after siRNA-mediated knockdown of *ADAR2* in human cardiomyocytes normalized to ß-actin (*n* = 3). **j** *CircAKAP13* expression after *ADAR2*-knockdown was analyzed using qRT-PCR (*n* = 6). **k** Amplified 3’AluSz sequence was analyzed using Sanger sequencing and then aligned to the hg38 genome. A-to-I (G) editing events were quantified and compared to the total adenosine content in sequence. Overexpression of *ADAR2* was validated on the **l** mRNA (*n* = 3) and on **m** protein level (*n* = 3). **n** Quantitation of ADAR2 protein expression after *ADAR2* overexpression in HEK293 cells normalized to ß-actin (*n* = 5). **o** *CircAKAP13* expression after *ADAR2* overexpression was analyzed using qRT-PCR (*n* = 3). Graphs show mean + SEM. Statistical differences were calculated using Student’s *t*-test. **p** Percentage of nucleotide mismatches in Alu elements after transfection with either *ADAR2* or a control plasmid. PCR products amplifying the 5’AluSx3 element were cloned and analyzed using Sanger sequencing. The sequence was aligned to the hg38 genome using UCSC’s BLAT. A-to-I (G) editing events were quantified and compared to the total adenosine content in the sequence. The level of **q** 5’AluSx3 and **r** 3’AluSz was determined using qRT-PCR 24 h after knockdown of *ADAR2* (*n* = 5). The level of **s** 5’AluSx3 and **t** 3’AluSz was determined using qRT-PCR 24 h after overexpression of *ADAR2* using qRT-PCR (*n* = 6). Graphs show mean + SEM. Statistical differences were calculated using Student’s *t*-test and Wilcoxon matched-pairs signed-rank test

ADAR2 protein levels and RNA editing events in Alu elements were significantly reduced in failing hearts. Therefore, we tested a potential interaction of ADAR2 with these Alu elements in *AKAP13*. RNA Immunoprecipitation of ADAR2 (Fig. 5a and b) showed a strong interaction with all three selected Alu elements flanking the *circAKAP13* BSS (Fig. 5c–f). We next investigated whether ADAR2 is involved in the formation of circRNAs in human cardiomyocytes. The reduction of ADAR2 led to an increase in *circAKAP13* (Fig. 5g–j) and other HF-regulated circRNA candidates (Fig. 3m), while reduction of ADAR1 did not affect *circAKAP13* (Supplementary Fig. 3c–e). The depletion of ADAR2 further resulted in a reduction of A-to-I RNA editing in the Alu element flanking the 3’ *circAKAP13* BSS (Fig. 5k). Accordingly, induction of ADAR2 led to a significant reduction in endogenous *circAKAP13* expression and an increase in A-to-I RNA editing in the flanking Alu element (Fig. 5l–p), while the *AKAP13* host gene mRNA was unchanged (Supplementary Fig. 5a). Thus, the interaction of ADAR2 with Alu elements regulates A-to-I RNA editing, thereby inhibiting *circAKAP13* RNA formation.

A-to-I RNA editing results in the conversion of AU base pairs to more unstable IU wobble base pairs that can destabilize dsRNA structures and lead to RNA degradation [64, 65]. Therefore, we analyzed the effects of ADAR2-mediated editing on the stability of Alu elements flanking the *circAKAP13* BSS. The reduction of ADAR2 resulted in increased levels of 5’AluSx3 and 3’AluSz (Fig. 5q and r). Conversely, ADAR2 over-expression resulted in the reduction of 5’AluSx3 and 3’AluSz levels (Fig. 5s and t). However, the level of 5’AluSz was not altered by ADAR2 modulation, which was in line with the unchanged level of A-to-G mismatches in HF (Fig. 4f, Supplementary Fig. 5b and c). These data suggest that RNA editing in Alu elements that can form a double strand may have an effect on the RNA stability of these Alu elements. Together, these data indicate that the process of circRNA formation in the human heart relies on the stability of flanking Alu elements, which is decreased by ADAR2-mediated A-to-I RNA editing.

### A-to-I editing in flanking Alu elements regulates circRNA formation

**Fig. 6:**
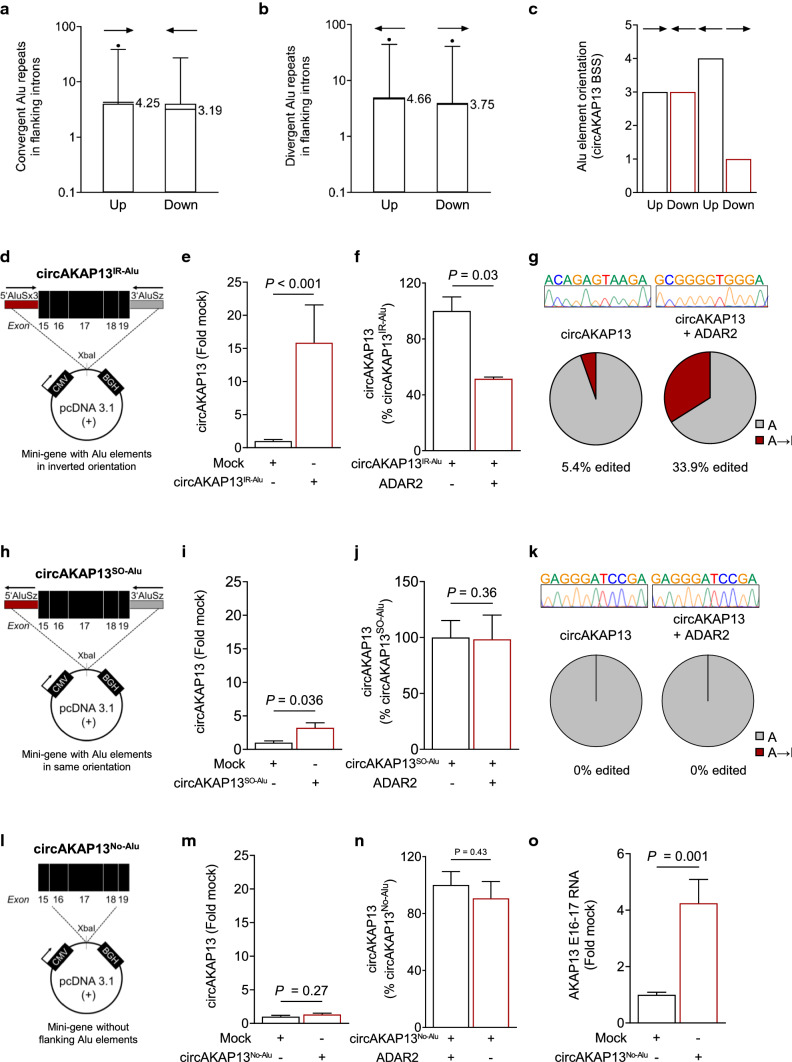
Impact of ADAR2 on RNA editing and circRNA formation using an *AKAP13* circRNA mini-gene with flanking Alu elements. Box-Whisker plot of **a** convergently and **b** divergently oriented Alu elements in flanking introns of HF-regulated circRNAs. Lines indicate mean (*n* = 173). **c** Alu elements with inverted orientation in the flanking introns of *circAKAP13* BSS, up indicates upstream and down indicates downstream. **d** Schematic depiction of a *circAKAP13* mini-gene with convergent Alu elements. A mini-gene containing the exons 15–19 of *AKAP13* mRNA as well as the flanking intronic 5’AluSx3 and 3’AluSz elements in convergent orientation was constructed and cloned into the pcDNA3.1 ( +) vector. IR-Alu indicates inverted repeated Alu elements. **e** *CircAKAP13* expression after transfection of the *circAKAP13* mini-gene (*n* = 5). **f** *CircAKAP13* expression was analyzed using qRT-PCR after co-transfection of *ADAR2* and the *circAKAP13* mini-gene (*n* = 3). Graphs show mean + SEM. Statistical differences were calculated using Mann–Whitney test. **g** Percentage of nucleotide mismatches in the 3’AluSz element after transfection with either the *circAKAP13* mini-gene alone or in a co-transfection with *ADAR2*. 3’AluSz PCR product was cloned and analyzed using Sanger sequencing. The sequence was aligned to the hg38 genome. A-to-I (G) editing events were quantified and compared to the total adenosine content in the sequence. **h** Schematic depiction of a *circAKAP13* mini-gene with Alu elements in the same orientation. A mini-gene containing exons 15–19 of *AKAP13* mRNA as well as the flanking intronic 5’AluSz and 3’AluSz elements in the same orientation was constructed and cloned into the pcDNA3.1 ( +) vector. SO-Alu indicates Alu elements in the same orientation. **i** *CircAKAP13* expression after transfection of the *circAKAP13* mini-gene into HEK293 cells using qRT-PCR (*n* = 4). **j** *CircAKAP13* expression was analyzed using qRT-PCR after co-transfection of *ADAR2* and the *circAKAP13* mini-gene (*n* = 4). Graphs show mean + SEM. Statistical differences were calculated using Student’s *t*-test. **k** Percentage of nucleotide mismatches in the 3’AluSz element after transfection with either the *circAKAP13* mini-gene alone or in a co-transfection with *ADAR2*. 3’AluSz PCR product was cloned and analyzed using Sanger sequencing. The sequence was aligned to the hg38 genome. **l** Schematic depiction of a *circAKAP13* mini-gene without Alu elements. A mini-gene containing exons 15–19 of *AKAP13* mRNA was constructed and cloned into the pcDNA3.1 ( +) vector. No-Alu indicates lack of Alu elements. **m** *CircAKAP13* expression after transfection of the *circAKAP13* mini-gene into HEK293 cells using qRT-PCR (*n* = 6). **n** *CircAKAP13* expression was analyzed using qRT-PCR after co-transfection of *ADAR2* and the *circAKAP13* mini-gene (*n* = 3). Graphs show mean + SEM. **o** Expression of AKAP13 mRNA between exons 16 and 17 after transfection with *circAKAP13* mini-gene into HEK293 cells measured by qRT-PCR (*n* = 6). Statistical differences were calculated using Student’s *t*-test

Next, we further elucidated the impact of ADAR2-mediated RNA editing on Alu elements flanking the circRNA BSS. In contrast to Alu elements in the same orientation, Alu elements flanking the BSS in either convergent or divergent orientation can promote circRNA formation [83]. Indeed, pairs of flanking Alu elements in inverted orientation are present in the majority of the identified HF-regulated circRNAs (Fig. 6a–c). We focused on *AKAP13* to determine the importance of the orientation of Alu elements. We designed two mini-genes containing exons 15–19, which are present in *circAKAP13*, as well as combinations of the flanking Alu elements. In the first mini-gene, the 5’AluSx3 and the 3’AluSz elements were chosen, based on their sensitivity to ADAR2 modulation and their convergent orientation (Fig. 6d). The transfection of this circRNA mini-gene resulted in enhanced levels of *circAKAP13*, whereas the mRNA expression of the host gene *AKAP13* was unchanged (Fig. 6e and Supplementary Fig. 5d and e). The simultaneous elevation of ADAR2 levels resulted in a reduction of *circAKAP13* and an increase in A-to-I editing in the flanking 3’AluSz element (Fig. 6f and g). Next, we wanted to investigate whether the formation of *circAKAP13* was exclusive to the combination of the flanking 5’AluSx3 and 3’AluSz elements in the convergent orientation. Therefore, we designed a second mini-gene containing the 5’AluSz and 3’AluSz elements, which are in the same orientation (Fig. 6h). The introduction of this mini-gene led to only a mild increase in the expression of *circAKAP13* (~ threefold, Fig. 6i) in contrast to the strong induction of *circAKAP13* (~ 15-fold) when Alu elements were in the convergent orientation (Fig. 6d and e). Interestingly, the simultaneous elevation of ADAR2 levels when using this *circAKAP13* mini-gene, with AluSz elements in the same orientation, did not result in a reduction of *circAKAP13* expression (Fig. 6j) nor editing (Fig. 6k). Furthermore, the complete lack of Alu elements did not result in elevated *circAKAP13* expression (Fig. 6l–o) highlighting the importance of Alu elements in *circAKAP13* formation. Together, these data reveal that ADAR2-mediated editing and repression of circRNAs in the human heart requires the presence of Alu elements in opposing directions.

### *CircAKAP13* overexpression in hiPSC-CM reduces sarcomere regularity

**Fig. 7:**
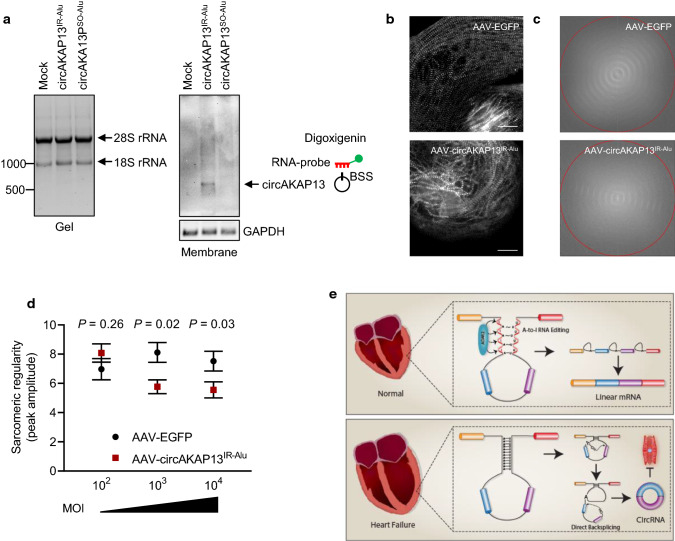
AKA13 mini-gene overexpression and its effect on sarcomere regularity. **a** Northern blot of circAKAP13 after overexpression of circRNA mini-genes with Alu elements. Total RNA was isolated after overexpression of circAKAP13 with inverted Alu element (IR-Alu), Alu elements in the same orientation (SO-Alu) or a control plasmid. **b** CircAKAP13-dependent effects in human iPSC-CMs were analyzed using AAV6-mediated overexpression of circAKAP13^IR−Alu^. Representative images after immunofluorescence staining of the sarcomeric protein titin M8/M9. Scale bar = 20 µm. **c** Sarcomeric structure was analyzed with fast Fourier transformation tool using ImageJ (FFT). Analysis is shown for representative pictures, in which hiPSC-CM were transduced with a MOI of 10^3^. **d** Analysis of sarcomeric regularity using FFT. Peak amplitudes were measured in hiPSC-CM after transduction with a MOI of 10^2^–10^4^ with AAV-circAKAP13^IR−Alu^ or AAV-GFP (*n* = 36–40 cells from *n* = 3 individual experiments). **e** Proposed mechanism of RNA editing and circRNA formation in failing human hearts. In the healthy human heart, ADAR2 maintains A-to-I editing in intronic Alu sequences governing canonical pre-mRNA splicing leading to mRNA. In the failing human heart, the ADAR2 protein is reduced resulting in reduced A-to-I editing in Alu elements. Alu repeats may pair and enhance the process of circularization resulting in direct back-splicing and the genesis of circular RNAs. Graphs show mean + SEM. Statistical differences were calculated using Student’s *t*-test

Finally, we assessed, whether the observed elevated *circAKAP13* levels in the failing human heart have functional consequences. Expression of the *circAKAP13* mini-gene containing Alu elements in convergent orientation led to an enhanced circRNA expression (Fig. 7a). Overexpression of this mini-gene in hiPSC-CMs using increasing multiplicities of infection (MOI) led to a significant reduction of sarcomere regularity at MOIs 10^3^ and 10^4^ particles per cell as determined by fast Fourier transformation (Fig. 7b–d). These data indicate a possible pathological function of circAKAP13 in the failing human heart. We propose that ADAR2-mediated A-to-I RNA editing represses the formation of dsRNA structures of Alu elements, thereby favoring canonical linear mRNA splicing and inhibiting the formation of circRNAs with pathophysiological consequences in the healthy human heart (Fig. 7e).

## Discussion

The post-transcriptional process of RNA editing is an important mechanism regulating gene expression, RNA stability and splicing to ensure cellular homeostasis [32, 39, 59, 62, 71]. The majority of these RNA editing events in the human heart occur in introns of protein-coding genes. We uncovered that over 99% of all detected RNA editing events in introns were in primate-specific Alu elements. ADAR2 contributes to A-to-I editing in these Alu elements, thereby repressing the formation of circRNAs, such as *circAKAP13*. In the failing human heart, ADAR2 is degraded, reducing RNA editing and increasing the formation of circRNAs.

A reduction in A-to-I RNA editing was not only found in cancer [9, 66] and neurological diseases [15, 41, 44, 52], but also in patients suffering from cardiac diseases [33]. There are only a few reported ADAR2-mediated editing events that result in codon changes of protein-coding genes [30, 33], whereas most ADAR2 editing sites are located in Alu elements of non-coding regions [74]. Consistently, we found the majority of A-to-I RNA editing in the human hearts occurs in Alu elements located in introns.

In this study, we identified protein-coding gene transcripts such as *RYR2* and *AKAP13* pre-mRNA as the most heavily edited in the human heart. Their editing was markedly reduced in failing human left ventricles. *AKAP13* is expressed in the heart and is essential for cardiac development [36, 72]. Here, we characterized the primate-specific process of circRNA formation and used the *AKAP13* gene to establish that circRNA formation is repressed by ADAR2-mediated RNA editing. The majority of cardiac circRNAs were independently regulated from their host genes, suggesting an alternative splicing mechanism. RNA editing in introns of protein-coding genes was associated with circRNA formation in a cancer cell line [32]. We found this mechanism to be conserved in the human heart, where we identified protein-coding genes with reduced RNA editing in introns giving rise to increased levels of circRNAs in the failing heart. Recent studies in the diseased heart found circRNAs to be up- and downregulated, whereas we found the majority of circRNAs to be increased [17, 40, 67, 73]. We found 16 of our 20 highest regulated circRNA candidates also upregulated in DCM in a recent study [73]. Heart failure is a heterogeneous disease, therefore we studied the regulation of circRNAs in a total of 20 patients with HF and 10 healthy controls. Finally, we validated differential regulation of circRNAs identified by RNA sequencing with diverged-primed qPCR. Various protective and regenerating functions have been attributed to circRNAs in the heart [80, 81, 84]. On the other hand, several circRNAs have been ascribed a pathological function, such as promoting cardiac senescence, enlarging infarct size post myocardial infarction or promoting hypertrophic cardiomyopathy and HF [18, 23, 38, 46, 49]. We identified the circRNA *circAKAP13* to be increased in HF patients. Overexpression of *circAKAP13* in hiPSC-CM resulted in decreased sarcomere regularity, suggesting a pathological function in the human heart. We describe the mechanistic cause of elevated circAKAP13 expression as reduced ADAR2-mediated A-to-I editing in the failing human heart.

Inverted Alu repeats can form RNA double strand structures serving as substrates for ADAR enzymes [42]. Notably, dsRNA stem-loop structures formed by inverted repeated Alu (IRAlus) elements have been associated with the formation of circular RNA (circRNA) [34, 48, 83]. Both ADAR enzymes are expressed in the human heart [3]. We observed an increase in ADAR1 and a reduction in ADAR2 in the failing heart. The reduction of ADAR2 and changes in the ADAR1/ADAR2 ratio have been reported in human diseases such as gastric cancer [10], glioblastoma [76] and congenital heart diseases [3]. This suggests a loss of ADAR2-mediated editing may be a general feature of human disease. Consistently, we saw a profound reduction in RNA editing in the heart muscle of HF patients. ADAR2 is essential for normal physiology, including postnatal development in mice, preventing seizures and neuronal death [30], and further protects from tissue ischemia [57]. Here, the data reveal a functional role for ADAR2-mediated RNA editing in intronic Alu elements, whereby ADAR2 inhibits circRNA formation in the human heart.

The levels of ADAR1 and ADAR2 protein expression are significantly altered in HF patients, while the mRNA expression levels are not affected. The enzymatic activity of the two ADAR enzymes is site-specific, only sharing a small subset of editing sites. Therefore, ADAR1 and ADAR2 cannot compensate for each other’s essential functions [8]. Our experiments show that ADAR2 undergoes proteasomal degradation in human cardiomyocytes. Interestingly, alterations in proteasomal degradation are associated with several heart diseases in human [79]. This is in line with recent reports in murine and human non-cardiac cells such as neurons, showing degradation of ADAR2 via the ubiquitin-dependent proteasome system [14, 27, 53]. The deletion of *Adar2* resulted in postnatal death in mice as a result of progressive seizures [30]. The impairment of ADAR2 was implicated in several neuronal diseases in humans such as neurodegenerative disorders [21, 30, 31, 45, 56], glioblastoma [76], gastric cancer [10], the fragile X syndrome, a genetic disorder associated with cardiac diseases [21, 69], cardiovascular disease [33], and congenital heart diseases [3]. A study from Prof. Junjie Xiao’s laboratory found that ADAR2 is increased in the trained mouse heart and protects against myocardial infarction and doxorubicin-induced cardiotoxicity [82]. Given the reduction of ADAR2, the profound reduction in A-to-I RNA editing and the increase of circRNA expression in the failing heart, we propose that ADAR2 is critically involved in the process of alternative circRNA splicing during the onset of HF.

ADAR2 binds to intronic Alu elements flanking BSS of *circAKAP13*, where we observed a strong reduction in A-to-I RNA editing in the failing heart. This finding is consistent with ADAR2 mediated A-to-I RNA editing of Alu elements in non-coding regions [70]. Mechanistically, we found that ADAR2-mediated regulation of circRNA splicing is based on the regulation of RNA stability of edited Alu elements. The observed mechanism relies on the orientation of the flanking complementary Alu elements, whereby convergent-orientated Alu elements, as observed in *AKAP13* introns, promote circularization efficiency. The mechanism we propose probably does not apply equally to all circRNAs. Thus, not all circRNAs were affected by ADAR2 reduction; in addition, a few circRNAs were also downregulated in the HF samples compared with the control samples. Presumably, additional mechanisms are responsible here, such as the presence, orientation, length, and distance of double-stranded Alu elements in the introns upstream and downstream of the BSS. In addition, mechanisms such as the binding of splicing enhancers or inhibitors known from classical alternative linear splicing are also potential factors affecting circRNA splicing. A conceivable therapeutic approach would be the modification of Alu elements using CRISPR/Cas system-based gene editing, which should lead to effects comparable to RNA editing and could thus have an influence on the genesis of circRNAs [7]. Further studies should show which circRNAs this mechanism applies to and whether this process is influenced by other factors, such as RNA-binding proteins.

In conclusion, we establish here that A-to-I RNA editing is the primary type of RNA editing in the human heart, predominantly occurring in introns of protein-coding genes and reducing the formation of circular RNAs. We found a profound reduction of A-to-I RNA editing in the failing human heart, concomitant with an increase in circRNA levels. The reduction of RNA editing in the failing heart is associated with a reduction in ADAR2, which modulates RNA stability of Alu elements and thereby controls circRNA formation as shown for *circAKAP13* via A-to-I RNA editing. The findings of our study are relevant to diseases with reduced RNA editing and increased circRNA levels and provide further insights into the human-specific regulation of circRNA formation.

## Supplementary Information

Below is the link to the electronic supplementary material.

Supplementary file1 (PDF 1090 KB)

Supplementary file2 (DOCX 50 KB)

## Data Availability

Sequencing data generated in this study and all other data supporting the findings of this study are available from the corresponding author upon reasonable request. Source data are provided with this paper.
