# Supplementary material for: Reduction of A-to-I RNA editing in the failing human heart regulates formation of circular RNAs
Source: Basic Res Cardiol. 2022 Jun 23;117(1):32. doi: 10.1007/s00395-022-00940-9 (PMC9226085; doi:10.1007/s00395-022-00940-9)

Supplementary Figures: Reduction of A-to-I RNA editing in the failing human heart regulates formation of circular RNAs

Basic Research in Cardiology

Karoline E. Kokot, MSc[1], Jasmin M. Kneuer, MSc[1], David John, PhD[2,3], Sabine Rebs, PhD[4-6], Maximilian N. Möbius-Winkler[1], Stephan Erbe[1], Marion Müller, PhD[7], Michael Andritschke[1], Susanne Gaul, PhD[1], Bilal N. Sheikh, PhD[8], Jan Haas, PhD[9,10], Holger Thiele, MD[11], Oliver J. Müller[12,13], Susanne Hille[12,13], Florian Leuschner, MD[9,10], Stefanie Dimmeler, PhD[2,3], Katrin Streckfuss-Bömeke, PhD[4-6], Benjamin Meder, MD[9,10], Ulrich Laufs, MD[1], Jes-Niels Boeckel, PhD[1*]

[1] Klinik und Poliklinik für Kardiologie, Universitätsklinikum Leipzig, Liebigstrasse 20, Leipzig, Germany.
[2] Institute for Cardiovascular Regeneration, Goethe-University Hospital, Theodor Stern Kai 7, Frankfurt, Germany.
[3] German Centre for Cardiovascular Research (DZHK), Partner site RheinMain, Frankfurt, Germany.
[4] Institute of Pharmacology and Toxicology, Versbacher-Str. 9, Würzburg, Germany.
[5] University Medicine Goettingen, Heartcenter – Clinic for Cardiology and Pneumology, Robert-Koch-Str. 40, Göttingen, Germany.
[6] German Centre for Cardiovascular Research (DZHK), Partner site Göttingen, Göttingen, Germany.
[7] Ruhr University of Bochum, Heart-and Diabetes Center North Rhine-Westphalia, Department of General and Interventional Cardiology/Angiology, Bad Oeynhausen, Germany.
[8] Helmholtz Institute for Metabolic, Obesity and Vascular Research (HI-MAG) of the Helmholtz Zentrum München at the University of Leipzig and University Hospital Leipzig, Leipzig, Germany.
[9] Department of Internal Medicine III, University of Heidelberg, Heidelberg, Germany.
[10] German Centre for Cardiovascular Research (DZHK), Partner site Heidelberg, Heidelberg, Germany.
[11] Heart Center Leipzig at University of Leipzig and Leipzig Heart Institute, Leipzig, Germany.
[12] University of Kiel, Dept. of Internal Medicine III, Kiel, Germany.
[13] German Centre for Cardiovascular Research (DZHK), partner site Hamburg/Kiel/Lübeck, Germany.

*Corresponding author:

Jes-Niels Boeckel, PhD

Klinik und Poliklinik für Kardiologie

Max-Bürger-Forschungszentrum (MBFZ)

Universitätsklinikum Leipzig

Johannisallee 30

04103 Leipzig

Boeckel@medizin.uni-leipzig.de

Supplementary Fig. 1

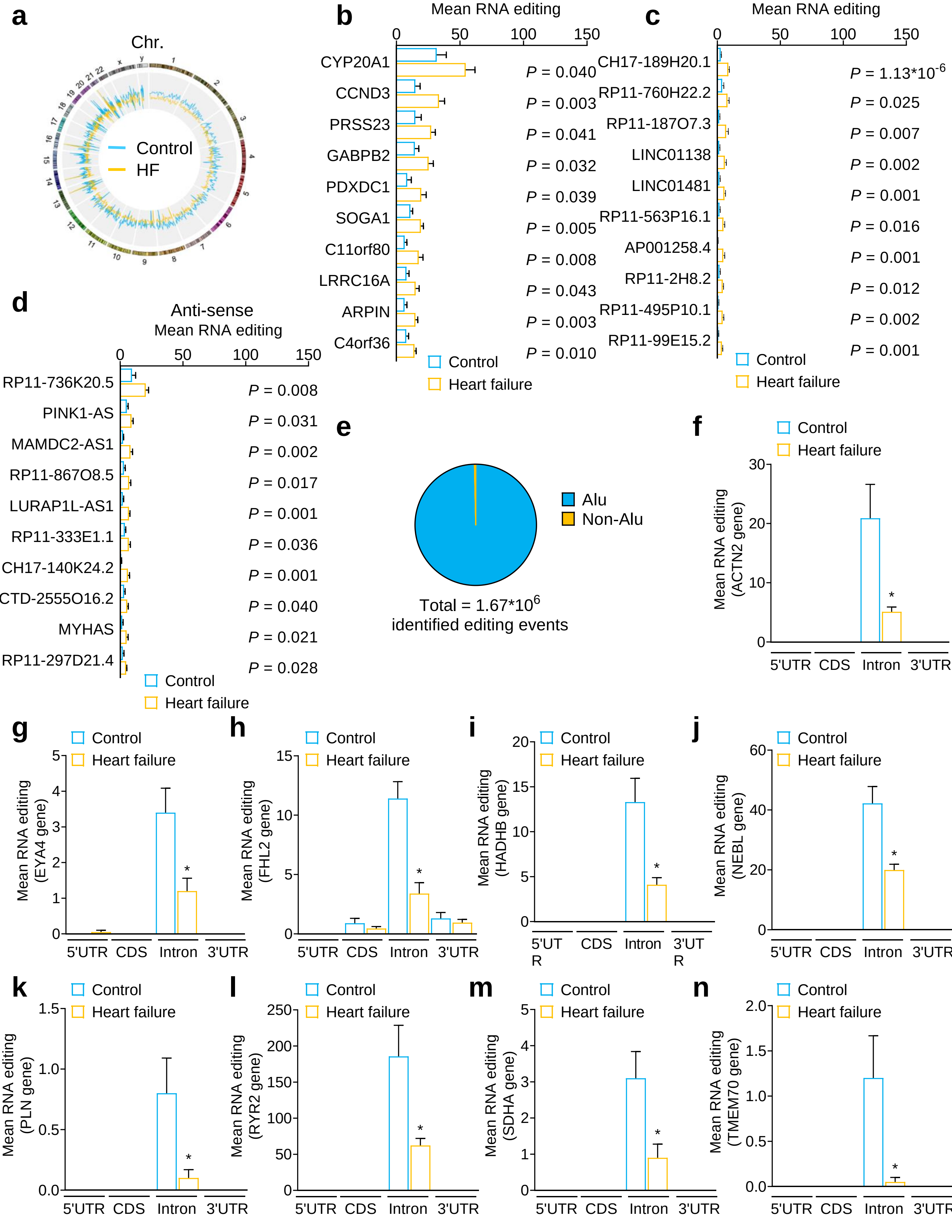

Supplementary Fig. 2

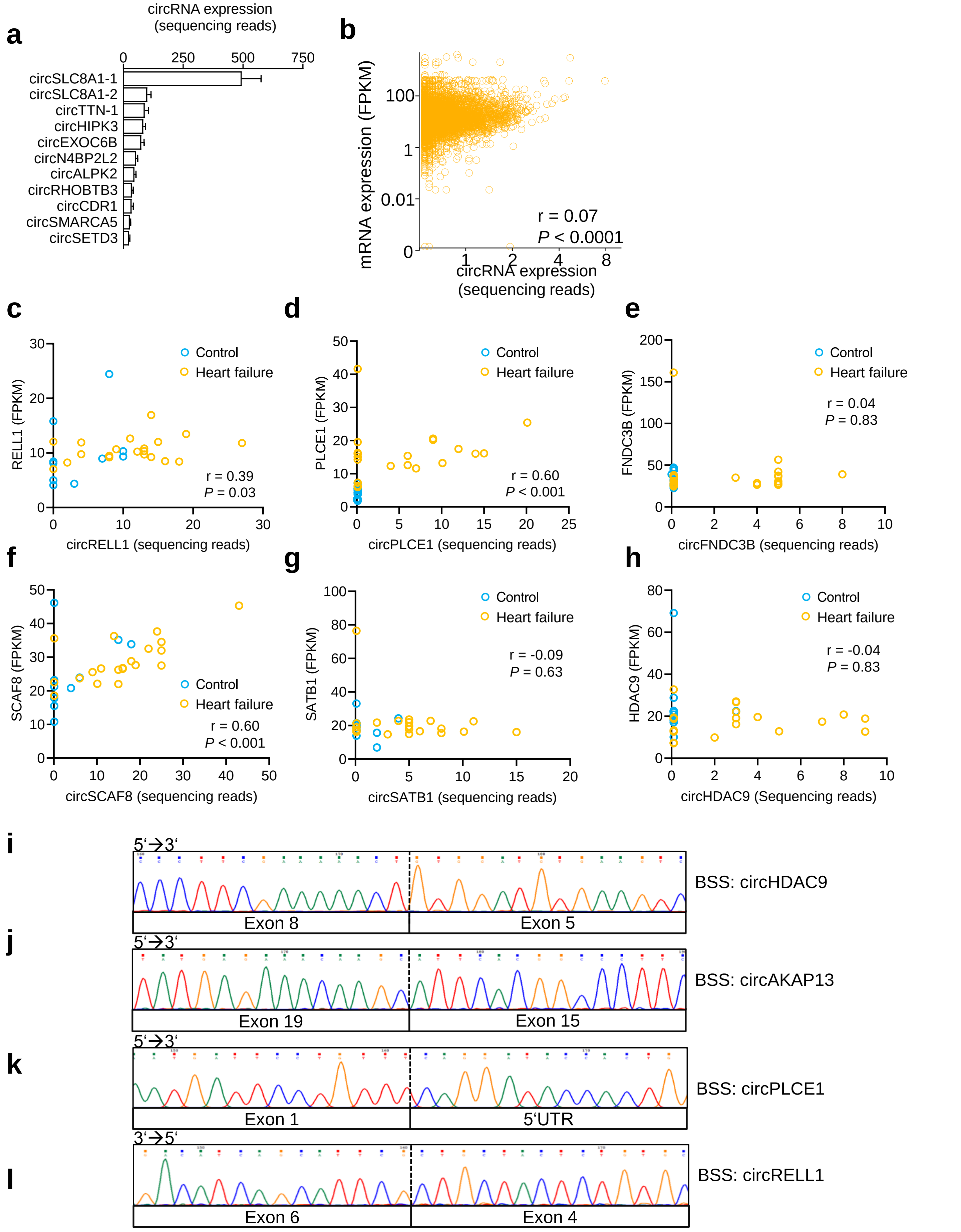

Supplementary Fig. 3

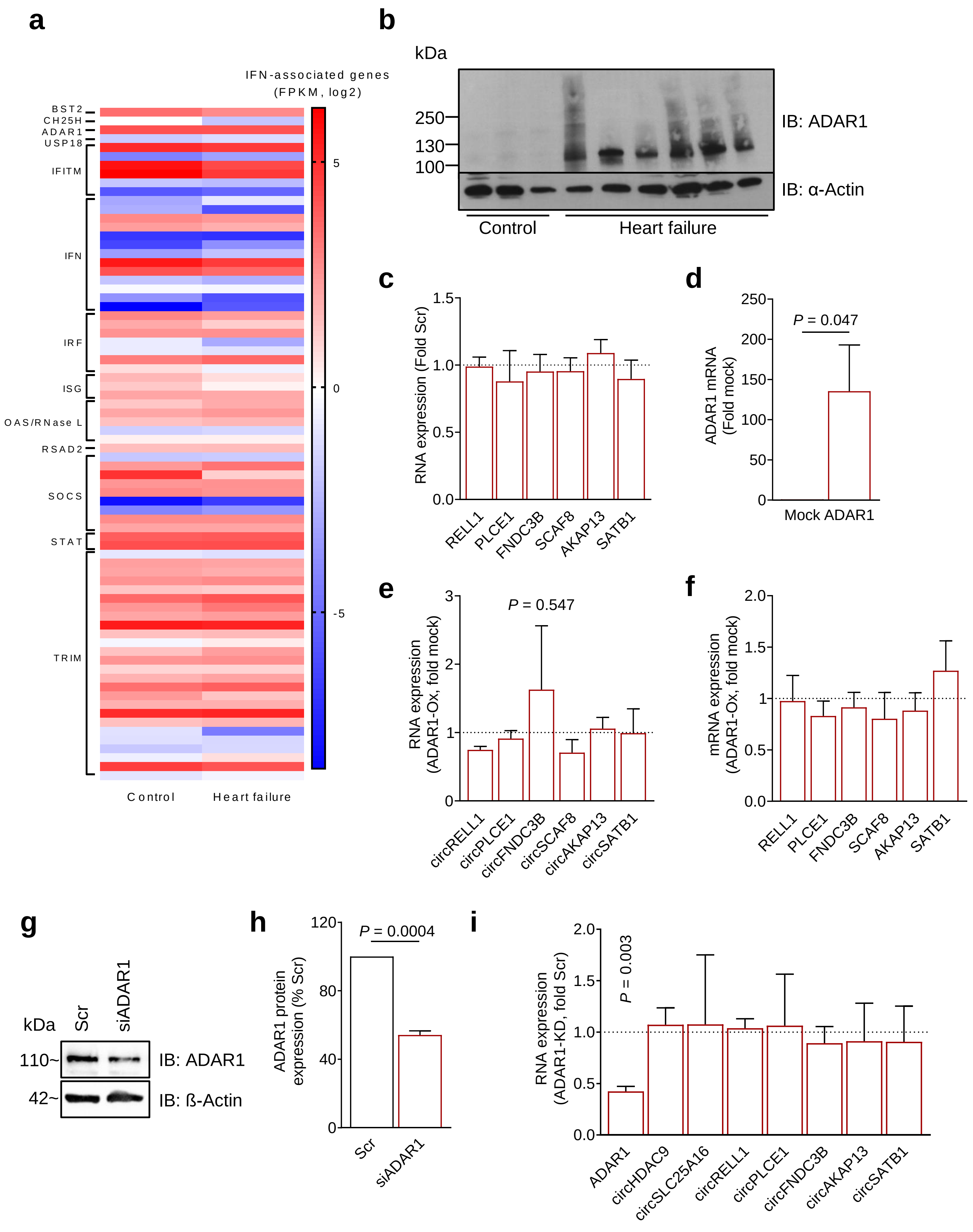

Supplementary Fig. 4

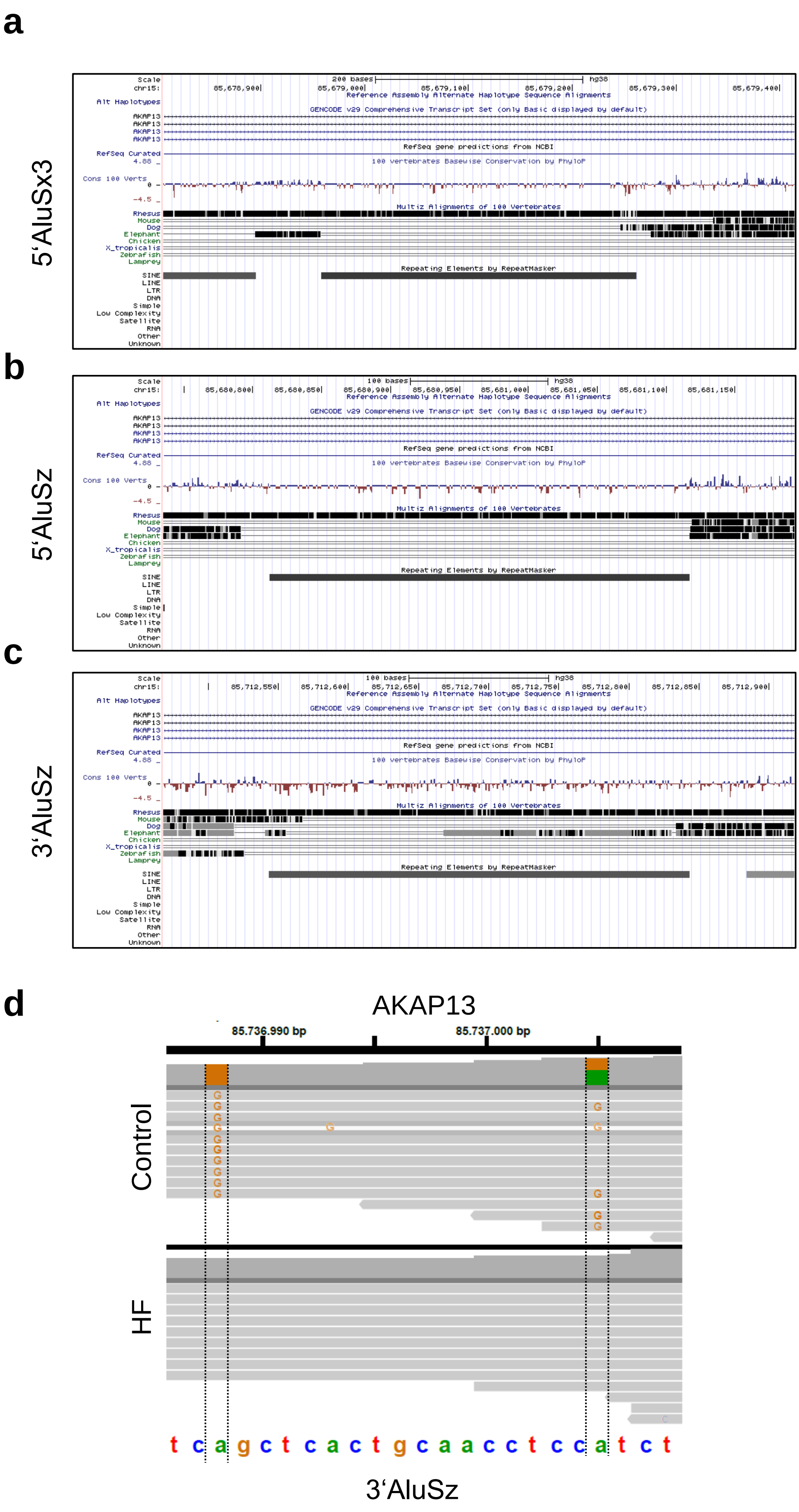

Supplementary Fig. 5

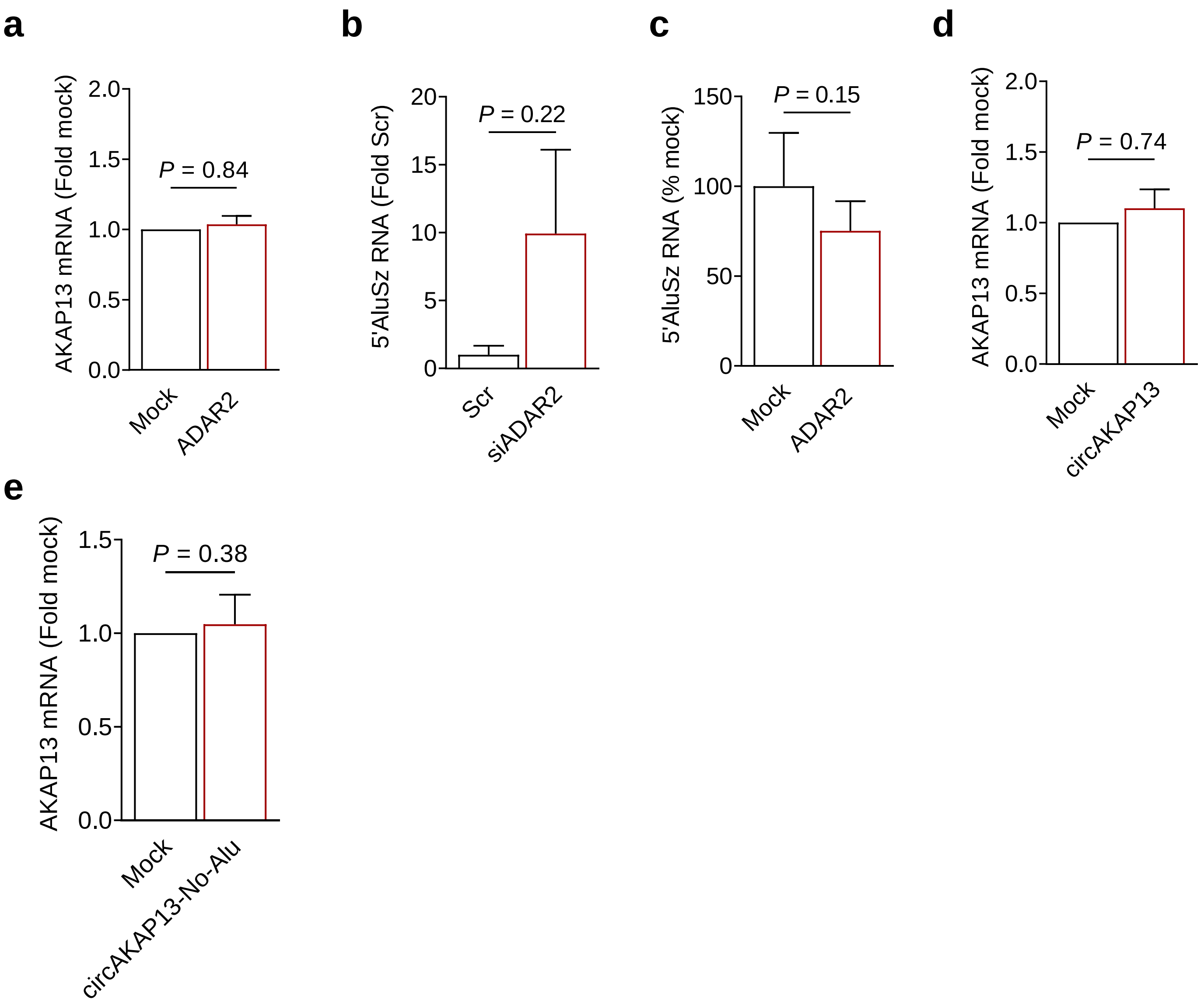

Supplement: Supplementary file 1 — Supplementary file1 (PDF 1090 KB) [file 395_2022_940_MOESM1_ESM.pdf]
