# Supplementary material for: Reduction of A-to-I RNA editing in the failing human heart regulates formation of circular RNAs
Source: Basic Res Cardiol. 2022 Jun 23;117(1):32. doi: 10.1007/s00395-022-00940-9 (PMC9226085; doi:10.1007/s00395-022-00940-9)
Supplement: Supplementary file 2 — Supplementary file2 (DOCX 50 KB) [file 395_2022_940_MOESM2_ESM.docx]

**SUPPLEMENTARY FIGURE CAPTIONS**

**Supplementary Fig. 1** RNA editing in human failing hearts. **(a)** Circos plot of genome-wide mean RNA editing events of control (blue, n=10) and failing (yellow, n=20) hearts. **(b)** Significant increase in RNA editing in protein-coding genes, **(c)** lncRNAs, and **(d)** anti-sense transcripts. **(e)** Total identified genome-wide RNA editing events grouped into repetitive Alu sequences and others. **(f-n)** RNA editing in selected HF-associated genes, grouped in the gene elements: 5‘UTR, coding sequence (CDS), intron, and 3‘UTR. **(f)** *ACTN,* **(g)** *EYA4,* **(h)** *FHL2,* **(i)** *HADHB,* **(j)** *NEBL,* **(k)** *PLN,* **(l)** *RYR2,* **(m)** *SDHA,* **(n)** *TMEM70* in failing hearts (n=20) and controls (n=10). All diagrams show mean + SEM. Statistical differences were calculated using Student‘s t-test

**Supplementary Fig. 2** Regulation and characterization of circRNAs in human failing hearts. **(a)** Highest expressed circRNAs in the failing and non-failing human heart determined by RNA sequencing and depicted as sequencing reads spanning the back-splice site (BSS). **(b)** Correlation of circRNA transcripts and their corresponding host genes expressions in human heart samples. Analysis was done after testing for normality using Spearman correlation. **(c-h)** Expression of selected circRNA transcripts correlated to their corresponding host gene mRNA using Spearman correlation. **(c)** *RELL1-circRELL1*, **(d)** *PLCE1-circPLCE1*, **(e)** *FNDC3B-circFNDC3B*, **(f)** *SCAF8-circSCAF8*, **(g)** *SATB1-circSATB1* and **(h)** *HDAC9-circHDAC9* expression in HF patients (n=20) and controls (n=10). **(i-l)** BSS from circRNA candidates validated by Sanger sequencing. **(i)** *circHDAC9,* **(j)** *circAKAP13,* **(k)** *circPLCE1,* and **(l)** *circRELL1*. CircRNA products were amplified from total RNA with PCR using divergent primers. PCR products were analyzed with Sanger sequencing to confirm the existence of specific circRNA BSS

**Supplementary Fig. 3 (a)** Heatmap describing mean log2 FPKM values of 76 interferon-associated genes in HF patients (n=20) and controls (n=10) taken from mRNA sequencing data. Interferon-associated genes were grouped by their respective gene family. **(b)** Immunoblot of ADAR1 protein expression in HF patients (n=6) and controls (n=3). This blot is also found in Figure 3d in a smaller portion**. (c)** Host gene mRNA expression of examined circRNA candidates (Fig. 3m) after siADAR2 knockdown. Human cardiomyocytes were transfected and RNA expression was analyzed with qRT-PCR after 24 h (n=3-4). **(d-f)** RNA expression after ADAR1 overexpression. **(d)** ADAR1 mRNA expression, **(e)** circRNA candidate expression and **(f)** host gene mRNA expression of circRNA candidates (n=3) 24h after transfection with ADAR1 plasmid. **(g-i)** RNA expression after siRNA-mediated knockdown of ADAR1 in HCM. **(g)** Representative immunoblot of siRNA-mediated ADAR1 knockdown in HCM, **(h)** quantitative analysis of ADAR1 protein levels 24 h after siRNA-mediated knockdown (n=4) and **(i)** *ADAR1* mRNA and circRNA fold changes of ADAR1 knockdown samples compared to controls (n=4). Graphs show mean + SEM**.** Statistical differences were calculated using Student‘s t-test

**Supplementary Fig. 4** RNA editing in intronic Alu sequences in the AKAP13 ORF. **(a-c)** Screenshots from the UCSC genome browser of **(a)** 5‘AluSx3, **(b)** 5‘AluSz, and **(c)** 3‘AluSz in proximity of the *circAKAP13* BSS showing the conservation of the sequences in different organisms. **(d)** An integrative map viewer screenshot of a 120 nt region of an Alu element upstream of the 5‘BSS of *circAKAP13* indicating the occurrence of RNA editing as determined by NGS data. The chromosomal region of representative control data (top), data from the failing heart (middle), individual RNA sequence reads (horizontal gray bars) and the *AKAP13* genomic DNA sequence (bottom) are shown. A-to-I (identified as G) nucleotide mismatches are indicated by the orange and green boxes. The dashed vertical lines highlight single nucleotide positions that underwent A-to-I editing

**Supplementary Fig. 5 (a)** *AKAP13* mRNA expression after ADAR2 overexpression (n=3). Statistical differences were calculated using Student‘s t-test. **(b-c)** 5’AluSz element stability in **(b)** HCM after knockdown of *ADAR2* (n=5) and **(c)** cells overexpressing ADAR2 (n=6). Statistical differences were calculated using Wilcoxon signed-rank test. **(d)** *AKAP13* mRNA expression after *circAKAP13-IR-Alu* overexpression (n=3). **(e)** AKAP13 mRNA expression after circAKAP13-No-Alu overexpression (n=6). Statistical differences were calculated using Student’s t-test

**SUPPLEMENTARY TABLE I: PRIMER SEQUENCES**

| Name | Sequence (F+R) | Remarks |
| --- | --- | --- |
| *RPLP0* | TCGACAATGGCAGCATCTAC | Reference gene |
|  | ATCCGTCTCCACAGACAAGG |  |
| *MALAT1* | GTCATAACCAGCCTGGCAGT | Control nucleus |
|  | AATCCCCTAGGGAAGGGGTC |  |
| *LaminB2* | AGCAGGAGATGACGGAGATG | Control Oligo(dT) |
|  | CATTCTCACGCATCACCGAG |  |
| *ADAR1* | CAATCAAGACACGGAGAGCC  TGGCTTTGCTGCTGAATTCA |  |
| *ADAR2* | GGCTCCACGAAAATGCTGAG |  |
|  | CGATTCCAGTGCGCTCCAAT |  |
| *AKAP13* | AGTTCTCTTCCGCTCCAACA |  |
|  | CCACATCTTGCTCAATGGGG |  |
| *PLCE1* | CCCCGTCAACAGTGGAGATA |  |
|  | ATCCTTCCATGGGCTGTCTC |  |
| *SCAF8* | CCTCCAGTTGTCACACCTGT |  |
|  | ACAGCCGCAAGTGTATCTGT |  |
| *RELL1* | ACACTGTTGGGCAAATCGTC |  |
|  | CTTTCAGGATCATACAGGCTGT |  |
| *FNDC3b* | CTCGTTCAAGTTAATCCAGGTGA |  |
|  | TGGGTGACATCATGGGAACT |  |
| *SATB1* | CTTGCAGTTACCGGAAGCTG |  |
|  | TAGCACGCTTCATTTCCTGC |  |
| *circAKAP13-1 (*hsa_circ_0036646) | CCTTCAGGCACATGACACATC |  |
|  | TCTCAGTCAGATTAGCACTGGT |  |
| *circPLCE1* | GAGCCAACGTCTGTCAGAAG |  |
|  | TGATCCATCACTGATTTGCAATGA |  |
| *circHDAC9* | ACACATTACCAGGAGCACAAG |  |
|  | GGTCTAAAGGTGAGATGGGCT |  |
| *circRELL1* | GAAACCGTCAATGGGGAGGT |  |
|  | TGTTATCTGCTACCATCGCCT |  |
| *circPDLIM5* | CTGAGCACATTCCAGCAGG |  |
|  | CTGAGGAGTTGCAGGAGGG |  |
| *circSLC25A16* | ACCCATTTGATGTGACTCGTC |  |
|  | TTTTGAGGAACAGCACGCAA |  |
| *circSLC8A1* | AGGACACTTGTGGAGAGCTC |  |
|  | CTCCCACCTTCCCCAAGTAG |  |
| *circASAP1* | ATTTTCTGGCTCCCTCGACT |  |
|  | TGTCCCCATCTTATGTGGCT |  |
| *circSATB1* | CGTGCTAAAGTGTCTCAAGCA |  |
|  | TGCCTCGTTCAAATGATCCA |  |
| *circFNDC3B* | GCACCAATTGACAACGGTTC |  |
|  | AGGCTTGCTGTACTGGTCTT |  |
| *circSDF4* | ATAGCGAGAAGGAGGTTGCC |  |
|  | TCTTCCGGTCAGTGTTCACA |  |
| *circSLOC5A1* | TCCCAAGTTCATCGAGTCACA |  |
|  | CCAAGTGCTCCCATGACATAC |  |
| *circSTK33* | TGTCCCTGATGCCAATTACAC |  |
|  | TCAATGACTATTCCAAAGCTCCC |  |
| *circSCAF8* | TGAGGGGACATAATCATCACTCA |  |
|  | GCTGCCTTAGTAATTTGGGTCA |  |
| *circAKAP13-2 (*hsa_circ_0104801) | GCCATTTCCTCTCCATTGACA |  |
|  | ATCGGTGGATGAACTGGATC |  |
| *circRYR2* | ATTTTCTGGCTCCCTCGACT |  |
|  | TGTCCCCATCTTATGTGGCT |  |
| *circTULP* | TCGCAGGGACAGGAGTACT |  |
|  | TACTCAGCTTGACCCAGGAC |  |
| *circTTN* | CCCGTTCCTGTTGCAAAGAA |  |
|  | GAGACCCACCGATTTTGCAT |  |
| *circDEK* | TGGAAGAAGTCACAATGAAACAGA |  |
|  | ACTTGCATTGTCAACCTCTCT |  |
| *circMLH3* | GAGGACATTCAGGCTGCTTG |  |
|  | GGCACTGGTTTCCTTCTCTG |  |
| GAPDH-DIG | CAGTCAGCCGCATCTTCTTT | Northern Blot |
|  | GGATCCTAATACGACTCACTATAGGTGACAAGCTTCCCGTTCTCA |  |
| circAKAP13-DIG | CCTTCAGGCACATGACACATC | Northern Blot |
|  | GGATCCTAATACGACTCACTATAGGTCTCAGTCAGATTAGCACTGGT |  |
| AKAP13-Ex16 | ATTTCCTGCCACATAGCCCC |  |
|  |  |  |
| AKAP13-Ex17  AluSx3 | GTGCCCGTTGACAGTCTTCT |  |
|  | GGAAGTAGCCAGGTCAGCAT |  |
| circAKAP13-DIG  AluSz14 | AGGGTTCCATTGTGTCGGAT |  |
|  | CATGGCGAGTCCCTGTTTCT |  |
| AKAP13-Ex16  AluSz20 | GTTCTACTACCAGACTACCAGCA |  |
|  | ACCTTTGATCCCCACAAGTATT |  |
| AKAP13-Ex17 | TGAGGCACAGAGAGGTTTGG |  |
| InfuAluSx3 | TGGCGGCCGCTCGAGTCTAGGGAAGTAGCCAGGTCAGC | Mini-gene construction |
|  | GGCCGTGAATAGGGTTCCATTGTGTCGG |  |
| InfucircAKAP13 | ATGGAACCCTATTCACGGCCCTTCCACAG | Mini-gene construction |
|  | GATCAAAGGTGCTTGTTTCTCATAATGACCGTGGG |  |
| InfuAluSz20 | AGAAACAAGCACCTTTGATCCCCACAAGT | Mini-gene construction |
|  | GGTTTAAACGGGCCCTCTAGTGAGGCACAGAGAGGTTTGG |  |
| InfuAluSz14 | TGGCGGCCGCTCGAGTCTAGACTACAGTTTTGGATTTCTGTTCA | Mini-gene construction |
|  | GGCCGTGAATCTCTGAGATTACTGTAAGCAAAACT |  |
| InfucircAKAP13 | AATCTCAGAGATTCACGGCCCTTCCACAG | Mini-gene construction |
|  | GATCAAAGGTGCTTGTTTCTCATAATGACCGTGGG |  |
| InfuAluSz20 | AGAAACAAGCACCTTTGATCCCCACAAGT | Mini-gene construction |
|  | GGTTTAAACGGGCCCTCTAGTGAGGCACAGAGAGGTTTGG |  |

**SUPPLEMENTARY TABLE II: ANALYZED CIRCRNAS AND THEIR CORRESPONDING CIRCBASE IDS.**

| Name | CircBase ID | Back-splice site (Chr:Base-Base) | p-value HF/ Control | Fold HF/Control |
| --- | --- | --- | --- | --- |
| circRELL1 | hsa_circ_0001400 | chr4:37631384-37638504 | 0.00192 | 2.8 |
| circSCAF8 | hsa_circ_0142309 | chr6:154773988-154833409 | 0.00139 | 3.5 |
| circPDLIM5 | No ID | chr4:94608058-94640450 | 0.00297 | 4.2 |
| circSLC25A16 | hsa_circ_0007778 | chr10:68487143-68506718 | 0.00217 | 4.7 |
| circSATB1 | hsa_circ_0064555 | chr3:18378169-18420991 | 0.00040 | 5.7 |
| circASAP1 | hsa_circ_0008934 | chr8:130152735-130180880 | 0.00201 | 5.8 |
| circSLC8A1 | No ID | chr2:40428472-40453349 | 0.00069 | 6.4 |
| circAKAP13-2 | hsa_circ_0104801 | chr15:85682157-85710645 | 0.00058 | 7 |
| circHDAC9 | hsa_circ_0001680 | chr7:18585280-18594029 | 0.00213 | 7.6 |
| circSDF4 | hsa_circ_0000002 | chr1:1223243-1223968 | 0.00256 | 7.9 |
| circTTN | No ID | chr2:178689812-178715774 | 0.00014 | 8.8 |
| circTULP1 | NO ID | chr6:158282262-158314268 | 0.00203 | 9.8 |
| circDEK | hsa_circ_0075796 | chr6:18236451-18258405 | 0.00179 | 11 |
| circSLCO5A1 | hsa_circ_0084727 | chr8:69738039-69761875 | 0.00015 | 11.6 |
| circRYR2 | hsa_circ_0112642 | chr1:237423091-237469187 | 0.00127 | 18.5 |
| circAKAP13-1 | hsa_circ_0036646 | chr15:85655416-85669830 | 0.00165 | 22.5 |
| circFNDC3B | hsa_circ_0003692 | chr3:172251259-172310881 | 0.00022 | 27.4 |
| circMLH3 | hsa_circ_0032649 | chr14:75046375-75049718 | 0.00179 | 30 |
| circSTK33 | hsa_circ_0096614 | chr11:8413494-8464822 | 0.00189 | 51.4 |
| circPLCE1 | hsa_circ_0019223 | chr10:94030682-94032252 | 0.00075 | 56.5 |
